# NQO1 protects obese mice through improvements in glucose and lipid metabolism

**DOI:** 10.1038/s41514-020-00051-6

**Published:** 2020-11-19

**Authors:** Andrea Di Francesco, Youngshim Choi, Michel Bernier, Yingchun Zhang, Alberto Diaz-Ruiz, Miguel A. Aon, Krystle Kalafut, Margaux R. Ehrlich, Kelsey Murt, Ahmed Ali, Kevin J. Pearson, Sophie Levan, Joshua D. Preston, Alejandro Martin-Montalvo, Jennifer L. Martindale, Kotb Abdelmohsen, Cole R. Michel, Diana M. Willmes, Christine Henke, Placido Navas, Jose Manuel Villalba, David Siegel, Myriam Gorospe, Kristofer Fritz, Shyam Biswal, David Ross, Rafael de Cabo

**Affiliations:** 1 Translational Gerontology Branch, National Institute on Aging Intramural Program, National Institutes of Health, Baltimore, MD 21224 USA; 2 Department of Environmental Health Sciences, Johns Hopkins Bloomberg School of Public Health, Baltimore, MD 21205 USA; 3 Nutritional Interventions Group, Precision Nutrition and Aging, Institute IMDEA Food, Crta. de Canto Blanco n° 8, 28049 Madrid, Spain; 4 Pharmacology and Nutritional Sciences, University of Kentucky College of Medicine, Lexington, KY 40536 USA; 5 Laboratory of Genetics and Genomics, National Institute on Aging Intramural Program, National Institutes of Health, Baltimore, MD 21224 USA; 6 Department of Pharmaceutical Sciences, Skaggs School of Pharmacy and Pharmaceutical Sciences, University of Colorado Anschutz Medical Campus, Aurora, CO 80045 USA; 7 Molecular Diabetology, Paul Langerhans Institute Dresden of the Helmholtz German Center for Diabetes Research Munich, University Hospital Carl Gustav Carus and Faculty of Medicine, TU Dresden, 01307 Dresden, Germany; 8 Centro Andaluz de Biología del Desarrollo, Universidad Pablo de Olavide-CSIC-JA, 41013 Sevilla, Spain; 9 Departamento de Biología Celular, Fisiología e Inmunología, Universidad de Córdoba, Campus de Excelencia Internacional Agroalimentario, ceiA3, Sevilla, Spain; 10 Present Address: Calico Life Sciences, South San Francisco, CA USA; 11 Present Address: University of Maryland School of Medicine, Baltimore, MD 21201 USA; 12 Present Address: College of Pharmaceutical Sciences and Chinese Medicine, Southwest University, Chongqing, 475004 People’s Republic of China; 13 Present Address: Harvard T.H. Chan School of Public Health, Boston, MA 02115 USA; 14 Present Address: Department Food Science, Cornell University, Ithaca, NY 14850 USA; 15 Present Address: Johns Hopkins Bloomberg School of Public Health, Baltimore, MD 21205 USA; 16 Present Address: Koch Institute for Integrative Cancer Research, Massachusetts Institute of Technology, Cambridge, MA 02139 USA; 17 Present Address: Emory University School of Medicine (MD/PhD program), Atlanta, GA USA; 18 Present Address: Department of Regeneration and Cell Therapy, Andalusian Center for Molecular Biology and Regenerative Medicine-CABIMER, Junta de Andalucia-University of Pablo de Olavide-University of Seville-CSIC, Seville, Spain

**Keywords:** Diabetes, Obesity

## Abstract

Chronic nutrient excess leads to metabolic disorders and insulin resistance. Activation of stress-responsive pathways via Nrf2 activation contributes to energy metabolism regulation. Here, inducible activation of Nrf2 in mice and transgenesis of the Nrf2 target, NQO1, conferred protection from diet-induced metabolic defects through preservation of glucose homeostasis, insulin sensitivity, and lipid handling with improved physiological outcomes. NQO1-RNA interaction mediated the association with and inhibition of the translational machinery in skeletal muscle of NQO1 transgenic mice. NQO1-Tg mice on high-fat diet had lower adipose tissue macrophages and enhanced expression of lipogenic enzymes coincident with reduction in circulating and hepatic lipids. Metabolomics data revealed a systemic metabolic signature of improved glucose handling, cellular redox, and NAD^+^ metabolism while label-free quantitative mass spectrometry in skeletal muscle uncovered a distinct diet- and genotype-dependent acetylation pattern of SIRT3 targets across the core of intermediary metabolism. Thus, under nutritional excess, NQO1 transgenesis preserves healthful benefits.

## Introduction

Diet-induced obesity is a risk factor for elevated cardiovascular mortality and type 2 diabetes (T2D), and a contributor to increased morbidities such as cancer, and chronic liver and kidney diseases. While 80% of individuals diagnosed with T2D are also obese, yet not all obese individuals develop this metabolic condition^1^. Family history, hyperglycemia, hypertension, unhealthy lipidomic profile, abdominal fat, and sterile inflammation, all contribute to individual susceptibility to T2D, suggesting that both genetic and metabolic factors are involved in its onset. Notwithstanding, the reason why obesity drives metabolic dysfunction in some but not all cases remains poorly understood.

The transcription factor nuclear factor erythroid-2-related factor 2 Nfe2l2 (also known as Nrf2) is a critical mediator of the cellular response to environmental stress. Under homeostatic conditions, Nrf2 is repressed by its tight association with the Kelch-like ECH-associated protein 1 (Keap1), which promotes Nrf2 ubiquitination and degradation through the ubiquitin-proteasome pathway. Exposure to a number of stressors, including electrophiles, reactive oxidative species, heavy metals, and xenobiotics leads to conformational changes in Keap1, enabling stabilization and nuclear accumulation of Nrf2, where it coordinates the expression of multiple cellular defense enzymes, such as NAD(P)H:quinone oxidoreductase 1 (NQO1), heme oxygenase 1 (HO-1), and key components of the glutathione (GSH) and thioredoxin (TXN) antioxidant systems^2^.

While the role of Nrf2 in protection from oxidative stress has been known for decades, its effect on metabolism is less clear. Activation of the Nrf2 pathway in *Keap1* hypomorphic mice can protect mice on HFD or genetically obese *db/db* mice from the development of obesity and T2D^3^. The mechanisms underlying these effects may involve protection of pancreatic beta-cells from oxidative damage^4^, repression of hepatic gluconeogenesis and lipid synthesis^5^, and regulation of muscle glycogen metabolism^6^. Unexpectedly, *Nrf2* knockout (KO) mice are protected against HFD-induced metabolic changes associated with type 2 diabetes phenotype^7,8^ despite their propensity to develop steatohepatitis. These results point to the potential cross-talk between Nrf2 and other metabolic pathways (reviewed in ref. ^9^).

The downstream target of Nrf2, NQO1, has also been implicated in protection against diabetes and metabolic syndrome. Metabolic syndrome is associated with increased reactive oxygen generation, leading to pro-inflammatory processes that ultimately elicit insulin insensitivity and the vicious cycle of the metabolic syndrome^10–12^. As an important enzyme in detoxification metabolism, NQO1 is rapidly induced in response to a variety of stressors^13^. NQO1 plays a direct role in protection from oxidative stress via its activity as a superoxide scavenger^14^. A null polymorphism in NQO1^15,16^ has been associated with an increased risk of complications related to metabolic syndrome^17,18^ and NQO1-null mice develop insulin resistance resulting in a T2D-like phenotype^19^. Pharmacological stimulation of NADH oxidation via NQO1-mediated catalysis has been associated with amelioration of both obesity^20^ and hypertension^21^ in mice, suggesting that NQO1 upregulation may be an effective therapeutic strategy for treating metabolic syndrome. However, NQO1 is also involved in other key cellular functions such as messenger RNA (mRNA) binding^22^, translation, and protein degradation by the 20S proteasome^23,24^. Furthermore, interaction with NQO1 is needed for maintaining the stability of several short-lived proteins, which include p53^25^ and the transcriptional coactivator PGC-1α^26^. By controlling mitochondrial biogenesis and respiration, both PGC-1α and p53 contribute to the cellular nutrient-sensing pathway. The ability of NQO1 to regulate the cellular NADH/NAD^+^ ratio could have repercussions in processes that depend on NAD^+^, including sirtuin-mediated regulation of mitochondrial proteins that protect against hepatotoxicity caused by nutrient excess^27–29^.

Here, we used genetic mouse models to explore the role of Nrf2 and its target gene NQO1 in conferring protection from diet-induced metabolic dysfunction. We show that Nrf2-mediated induction of NQO1 leads to improved glucose homeostasis in *Keap1*-KO mice consuming a western diet. These results led us to directly examine the benefits of NQO1 transgenesis (NQO1-Tg), and its contribution to improved insulin sensitivity and metabolic dysfunction by performing an integrated analysis of physiological data, histological changes, and “omics” profiles from serum and metabolically active tissues such as liver, skeletal muscle, and visceral fat in mice fed either standard diet (SD) or high-fat diet (HFD). Our results show that NQO1 overexpression leads to improvements in insulin sensitivity and in glucose, lipid and NAD^+^ metabolism while providing protection from HFD-induced liver steatosis and macrophage infiltration in the adipose tissue. We show that these effects are mediated by attenuation of signaling downstream of mTORC1, as evidenced by reduced S6K1 phosphorylation, and elevation of 4E-BP1. Taken together, these findings shed light on a previously unappreciated cross-talk between redox enzymes and mTORC1 signaling pathways and provide support for selectively targeting NQO1 in metabolic conditions.

## Results

### Whole-body deletion of *Keap1* improves insulin sensitivity and upregulates NQO1 expression

**Fig. 1:**
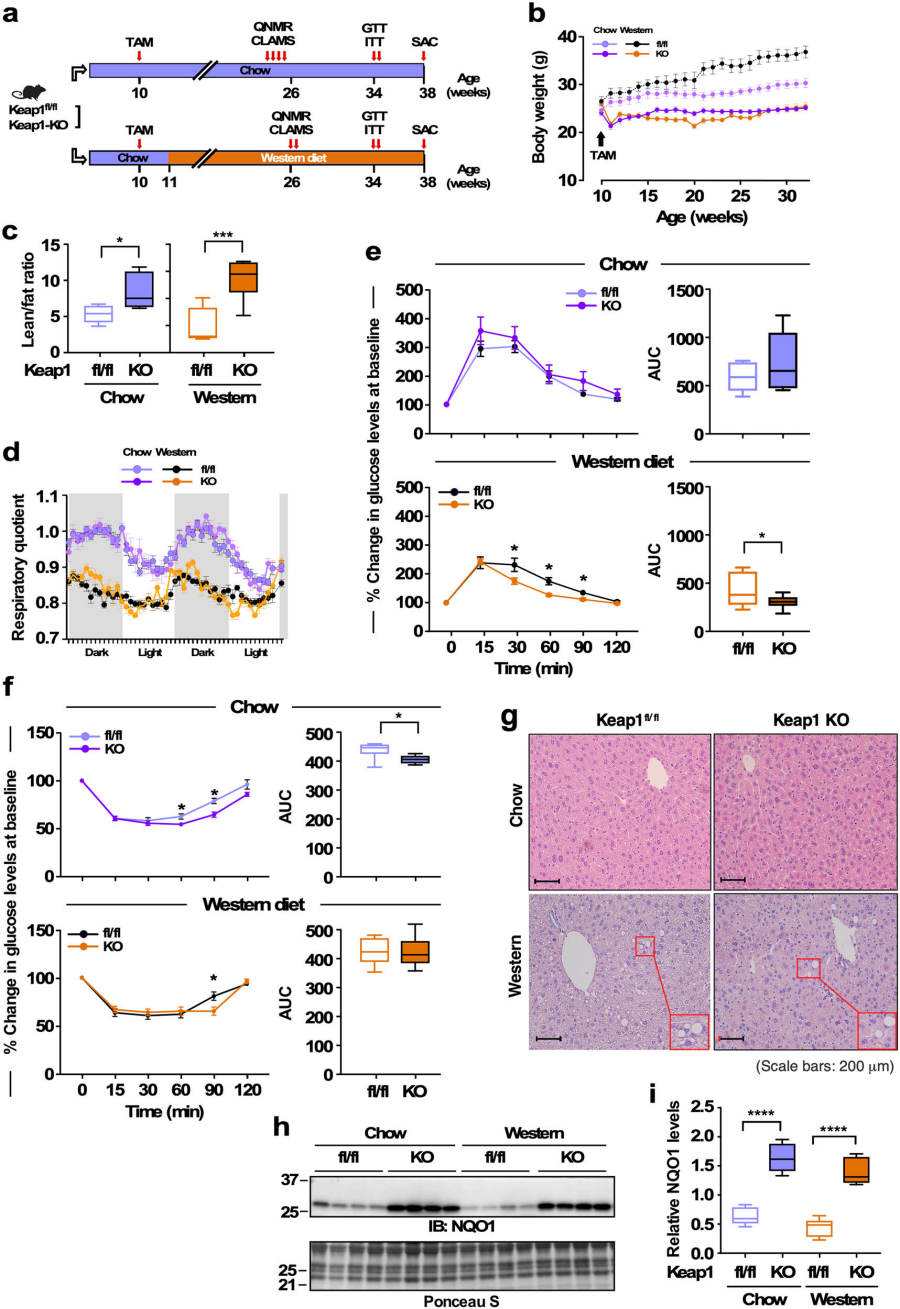
Effect of global Keap1 deletion and diet composition on markers of health in mice. **a** Diagram representing the experimental protocol. **b** Body weight trajectories over the course of 22 weeks after tamoxifen (TAM) administration. *n* = 8–10 per group. **c** Lean-to-fat ratio calculation for mice on regular chow and on a western diet. *n* = 6–8 per group. **d** Mice were placed into metabolic cages for 48 h to measure the respiratory exchange ratio (RER). *n* = 6–7 per group. **e** Left panels, trajectories of blood glucose clearance during an oral glucose tolerance test as percent change from baseline; right panels, area under the curve (AUC). *n* = 5–10 per group. **f** Left panels, trajectories of blood glucose clearance during an insulin tolerance test as percent change from baseline; right panels, area under the curve (AUC). *n* = 6–10 per group. **g** H&E staining of fixed liver tissues. **h** Western blot for NQO1 in skeletal muscle extracts. Molecular mass markers (kDa) are depicted on the left. **i** Densitometric quantification after normalization with Ponceau S staining of the membrane. *n* = 6–8 per group. All data are expressed as means ± SEM. Comparison by two-tailed *t*-test was performed unless otherwise specified. *, ***, ****p < 0.05, 0.001, and 0.0001 vs. fl/fl littermate controls.

To investigate the role of *NRF2* hyperactivation in metabolism, we generated a transgenic mouse strain exhibiting whole-body inducible knockout of the *Keap1* gene, by crossing *Keap1* floxed mice^30^ with mice expressing Cre recombinase under the control of the CAG promoter (Keap1-KO; *Keap1*^*fl/fl*^; CreERT2^+^). Tamoxifen treatment was initiated in 3-mo-old male mice at a time when maturational growth rate has nearly plateaued (https://www.jax.org/research-and-faculty/research-labs/the-harrison-lab/gerontology/life-span-as-a-biomarker). The effect of *Keap1* deletion was examined first by performing morphometric and physiological analyses at various timepoints (Fig. 1a).

Over the course of 20 weeks of treatment, Keap1^fl/fl^ mice on ad libitum standard chow (14% fat, 54% carb., 32% protein) and western diet (40% fat, 43% carb., 17% protein) increased their body weight by 22% and 36%, respectively (31.0 ± 0.8 g vs. 24.6 ± 1.1 g, *p* < 0.0005 (*n* = 7); 37.2 ± 1.3 g vs. 26.5 ± 1.0 g, *p* < 0.0001 (*n* = 8)), while mice in which Keap1 has been deleted did not gain significant weight regardless of the diet (Fig. 1b). Both fl/fl and KO groups of mice consumed the same amount of food daily throughout the experiment (g/mouse/day; chow: 3.19 ± 0.03 [fl/fl], 3.27 ± 0.03 [KO]; western diet: 2.67 ± 0.04 [fl/fl], 2.61 ± 0.06 [KO], mean ± SEM). This phenotype correlated with considerable improvement in the lean-to-fat ratio regardless of diet, with KO mice exhibiting significantly lower percentage of body fat and greater lean mass than their littermate controls, especially in mice fed an obesogenic diet (Fig. 1c and Supplementary Fig. 1a).

In vivo metabolic differences were found in response to diet type rather than the presence or absence of *Keap1* (Fig. 1d). Respiratory quotient fluctuations, calculated from the amount of CO_2_ produced over O_2_ consumed between the dark and light cycles, were clearly higher in mice on regular chow than those fed a western diet, indicating greater metabolic flexibility in chow-fed animals.

The effect of *Keap1* deletion on glucose homeostasis and insulin sensitivity was then examined by performing both glucose tolerance and insulin tolerance tests (GTT and ITT, respectively). Compared to *fl/fl* littermates, KO mice exhibited improved glucose clearance with both diet types as evidenced by reduced area under the curve (AUC) (Supplementary Fig. 1b) and lower fasting glucose levels (28% or 44% reduction when fed standard chow or western diet, respectively) (Supplementary Fig. 1c). These beneficial outcomes on glucose disposal were preserved only in KO mice on western diet after adjustment for baseline blood glucose (Fig. 1e). However, insulin sensitivity in mice lacking *Keap1* was slightly improved when fed standard chow, i.e., lower AUC values, with a less pronounced effect under obesogenic diet (Fig. 1f). These data suggest that global *Keap1* deletion results in body weight loss associated with fat mass reduction, return of fasting blood glucose within normal ranges, and improvement in glucose tolerance. Significant increases in muscle mass (quadriceps and gastrocnemius) were observed in *Keap1*-KO mice fed a western diet vs. obese *fl/fl* littermates (Supplementary Fig. 1d), with coincident reduction in hepatic steatosis in KO animals (Fig. 1g).

We next ascertained whether mice lacking Keap1 would exhibit higher constitutive Nrf2 activation than *fl/fl* littermate controls. Immunoblotting of skeletal muscle extracts of KO mice revealed a diet-independent upregulation of NQO1 protein (Fig. 1h, i). In contrast, loss of Keap1 did not have a substantial impact on superoxide dismutase 2 (SOD2) expression while eliciting a decrease in HO-1 levels in KO mice on western diet (Supplementary Fig. 1e, f). Deletion of *Keap1* was effective at upregulating the levels of glutamate-cysteine ligase catalytic subunit (GCLC) in muscle of KO mice fed either chow or western diet (Supplementary Fig. 1e, f), although to levels much lower compared to NQO1 (Fig. 1h, i). Like the pattern observed in skeletal muscle, expression of NQO1 along with other three targets of Nrf2 were altered in KO vs. *fl/fl* mouse liver (NQO1»GCLC > SOD2 = HO1; Supplementary Fig. 1g, h). Because of the low amount of fat tissue present in KO mice, we only performed quantitative PCR analysis and found significant upregulation of NQO1 and GCLC mRNA with Keap-1 deletion regardless of the diet, whereas no changes in HO1 and SOD2 mRNA were detected (Supplementary Fig. 1i).

Altogether these data indicate that NQO1 exhibited major quantitative changes demonstrating its importance as a key downstream target of *Nrf2* in several *Keap-1*^*fl/fl*^ mouse tissues involved in glucose homeostasis.

### NQO1 overexpression improves glucose homeostasis and insulin sensitivity in Tg mice on HFD

**Fig. 2:**
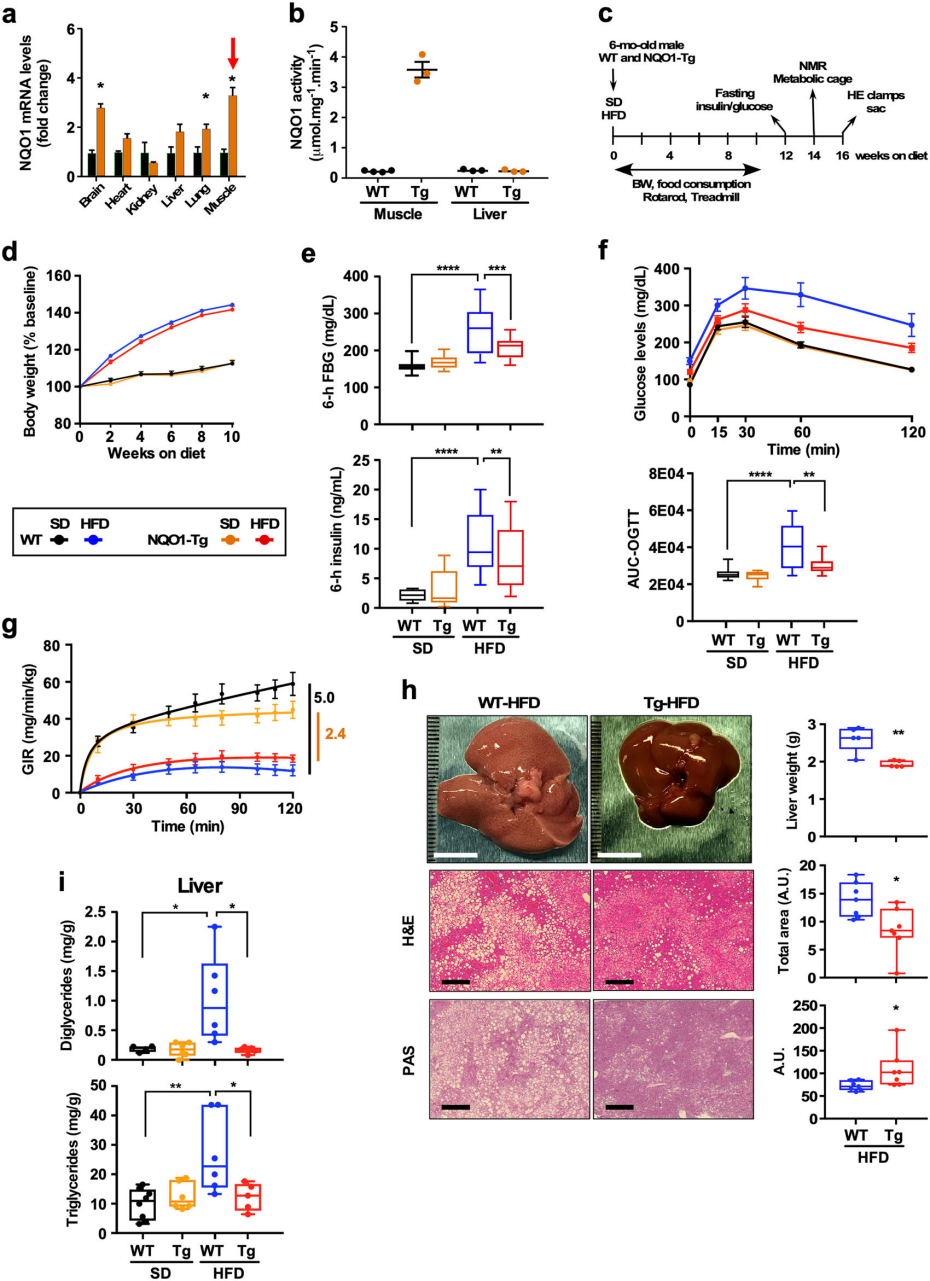
Effect of global NQO1 transgenesis on body weight, glucose metabolism, insulin sensitivity, and liver steatosis in mice-fed SD and HFD diet. **a** Expression patterns of NQO1 mRNA in tissues of WT (green bars) and NQO1-Tg (orange bars) mice. Red arrow shows enhanced NQO1 mRNA levels in skeletal muscle of transgenic mice. *p < 0.05 vs. littermate controls. **b** Enzymatic activity of NQO1 in muscle and liver extracts of WT and NQO1-Tg mice (*n* = 3–4 per group). **c** Timetable for the measure of the indicated parameters in male WT and NQO1-Tg mice. **d** Trajectories of body weight gain expressed as percent of baseline. Values are means ± SEM, *n* = 22–36 mice per group. **e** Fasted blood glucose (FBG) and insulin levels after a 6-h fast. Values are represented as boxplots with *n* = 9–17 mice per group. **f** *Upper panel*, Oral glucose tolerance test (OGTT) performed on mice fed either SD or HFD for 12 weeks. Values are means ± SEM, *n* = 10–12 mice per group. *Lower panel*, Area under the curve (AUC) values are represented as boxplots. **g** Hyperglycemic-euglycemic clamp experiment was performed to study insulin action in mice. Values are means ± SEM, *n* = 10 mice per group. **h** *Left panels*, Visual inspection of the appearance of the liver of mice-fed HFD for 16 weeks (top), bar = 1 cm; fixed liver tissue from HFD-fed mice were H&E stained (middle) or processed for periodic acid-Schiff (PAS) stain (bottom). Each specimen was examined at the same magnification. *Right panels*, liver weight and accumulation of lipid droplets and glycogen using H&E and PAS stained sections, respectively, *n* = 6–7 per group. **i** Levels of diglycerides and triglycerides in the liver of WT and NQO1-Tg mice-fed SD and HFD, *n* = 4–8 per group. *, **, ***, ****p < 0.05, 0.01, 0.001, 0.0001.

**Fig. 3:**
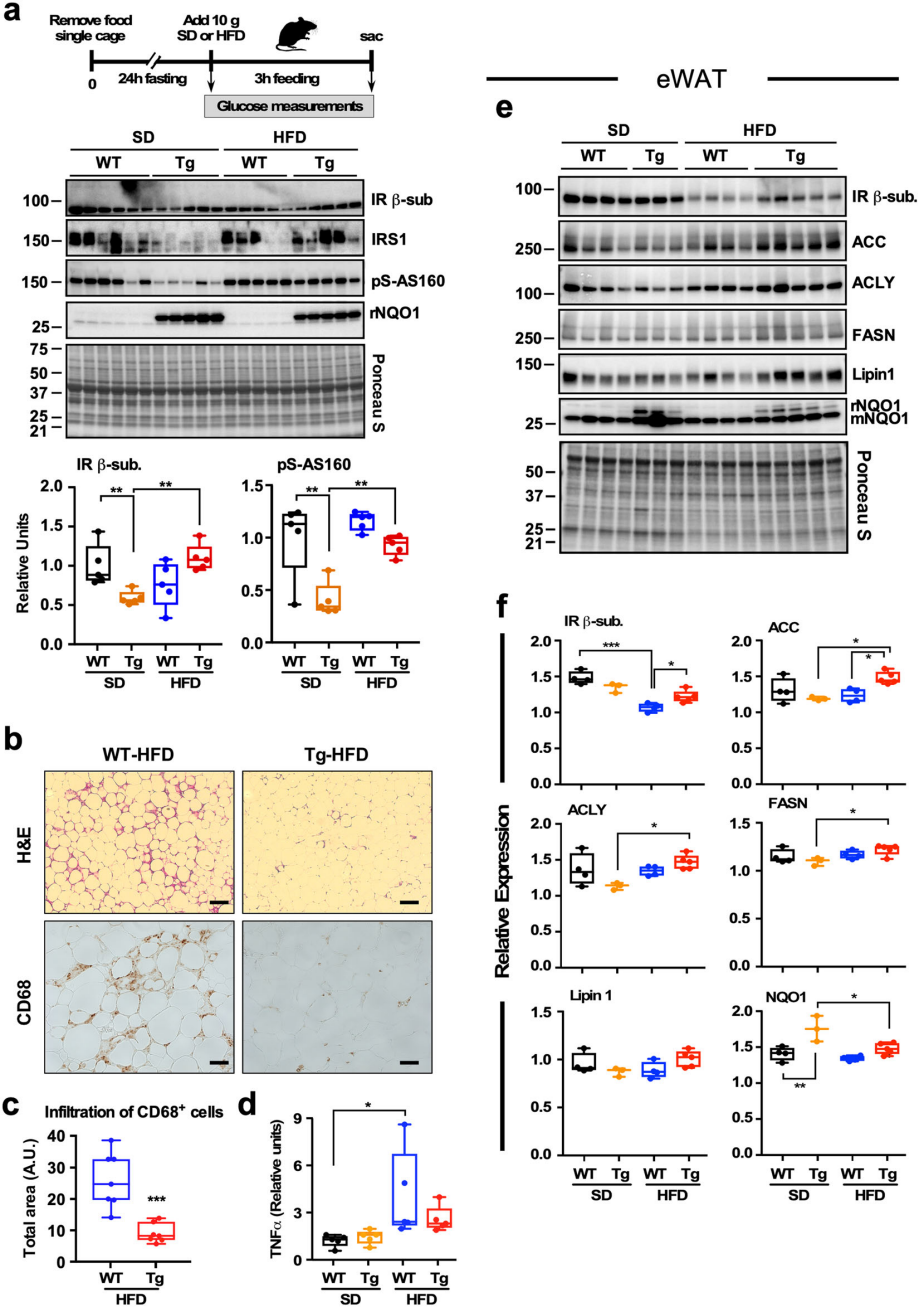
Effect of global NQO1 transgenesis and diet composition on key players of insulin action in skeletal muscle and eWAT of mice. **a** Comparison of the 3-h refeeding period with SD and HFD on insulin receptor/IRS-1 pathway activation. Upper diagram illustrates the experimental protocol. *Middle panel*, immunoblots for IR β-subunit, IRS-1, serine phosphorylated AS160, and NQO1 from skeletal muscle homogenates. Note the marked accumulation of rat NQO1 transgene. Ponceau S staining of the membrane is shown and the molecular weight protein standards (kDa) are depicted on the left. *Bottom panel*, densitometric quantification of IR β-subunit and phosphorylated AS160. Values are represented as boxplots with individual values (*n* = 5 per group). **b** Fixed adipose tissue from HFD-fed mice were H&E stained (top panels) and processed for expression of CD68 by immunohistochemistry (bottom panels). Each specimen was examined at the same magnification. **c** Degree of fat cell infiltration by CD68^+^ cells, *n* = 7. **d** Relative TNFα mRNA levels in the liver determined by quantitative PCR, *n* = 4–5. **e** Immunoblots for IR β-subunit, ACC, ACLY, FASN, Lipin 1, and NQO1 from eWAT homogenates. Note the presence of the rat NQO1 transgene. **f** Densitometric quantification of the immunoblots depicted in **e**, *n* = 3–5. *, **, ***p < 0.05, 0.01, 0.001.

To test the hypothesis that upregulation of NQO1 ameliorates diet-induced imbalance in metabolic homeostasis, we generated a transgenic mouse strain that constitutively over-expresses the rat NQO1 gene under the cytomegalovirus promoter (NQO1-Tg) (see Experimental model details in Methods for additional information). Although mouse and rat NQO1 have the same length (274 amino acids) and their sequences are 93.8% identical, we capitalized on the fact that the rat NQO1 migrates at a higher molecular weight on sodium dodecyl sulfate polyacrylamide gel electrophoresis (SDS-PAGE)^31^, making it distinguishable from the endogenous murine form. Preliminary experiments confirmed the significant increase in NQO1 mRNA levels in several tissues of Tg mice, including brain, lung and skeletal muscle, but not kidney, heart or liver (Fig. 2a). Immunoblotting further confirmed accumulation of the rat isoform of NQO1 in skeletal muscle (Fig. 3a), epididymal WAT (eWAT, Fig. 3e) and pancreas (Supplementary Fig. 2a), but not liver (Supplementary Fig. 2a). Epigenetic silencing of the transgene may have contributed to some tissues not having increased expression of rat NQO1. NQO1 enzymatic activity was induced 15-fold in muscle extracts of Tg mice vs. littermate WT controls (Fig. 2b). These results show a key difference between the *Keap-1*^*fl/fl*^ and NQO1-Tg mouse models, with overexpression of endogenous NQO1 protein in the liver of Keap1-KO and the lack of expression of the rat transgene in NQO1-Tg liver.

To better define the impact of NQO1 transgenesis on physiological outcomes, we investigated 6-mo-old male NQO1-Tg mice and littermate controls (WT) that were fed either a standard laboratory diet (SD) or a high-fat diet (HFD, 60% kcal fat) ad libitum for periods up to 16 weeks (Fig. 2c). Although responsive to diet type, NQO1-Tg mice had similar weight gain trajectories (Fig. 2d) and consumed the same amount of food (Supplementary Fig. 2b) as their WT littermates. Compared to SD, mice on HFD for 14 weeks had significantly lower lean-to-fat ratios regardless of the genotype (Supplementary Fig. 2c). However, the surge in fasting blood glucose (FBG) and insulin levels in WT mice on HFD was significantly reduced in HFD-fed Tg mice, consistent with improved organismal metabolic homeostasis upon NQO1 overexpression (Fig. 2e). To determine whether the changes in FBG reflected differences in glucose homeostasis, we conducted OGTT on 6-h fasted mice after 12 weeks on either SD or HFD. Glucose clearance was indistinguishable between Tg and WT mice on SD, while for HFD-fed Tg mice the rate of glucose disposal was significantly faster than in WT-HFD mice, with AUC in Tg mice approaching the glucose clearance rate observed under SD (Fig. 2f and Supplementary Fig. 2d).

To determine whether NQO1 transgenesis elicited beneficial effects on insulin sensitivity in vivo, we performed hyperinsulinemic–euglycemic clamp after 16 weeks on diet (Fig. 2g). The glucose infusion rate (GIR) decreased dramatically ~5-fold in WT mice on HFD compared to SD (in mg/min/kg, from 58.9 ± 6.2 to 12.6 ± 3.1, *p* < 0.0001) and the time to reach euglycemia was also significantly shorter (in min, from 57.5 ± 7.0 to 89.0 ± 7.5, *p* = 0.007). In contrast, for Tg mice HFD feeding resulted in less than 2.4-fold reduction in GIR compared to SD (in mg/min/kg, from 44.6 ± 5.1 to 18.4 ± 1.7, *p* < 0.0001) with comparable time to reach euglycemia (in min, from 74.0 ± 10.7 to 70.5 ± 10.1, *p* = 0.813). No differences in organ weight (eWAT, liver, quadriceps femoris) were observed between WT and NQO1-Tg mice on HFD for 16 weeks (Supplementary Fig. 2e). Neuromuscular function was assessed in a separate cohort of mice. Animals (18 weeks of age) were put on HFD for 14–15 weeks prior to testing. Coordination and treadmill endurance were found to be significantly reduced in WT mice on HFD, while the same measures were largely preserved in NQO1-Tg mice (Supplementary Fig. 2f).

Regardless of the diet and genotype, diurnal rhythmicity in VO_2_, VCO_2_, locomotion, and heat production was observed when measured using the Comprehensive Laboratory Animal Monitoring System (CLAMS) (Supplementary Fig. 2g). Consistent with previous reports that ad libitum-fed mice on standard laboratory chow consume a mix of carbohydrates and fat, we observed that respiratory exchange ratio (RER) fluctuated daily between 0.80 and ~0.85 when fed SD and remained mostly at ~0.70 in HFD-fed mice independent of the genotype (Supplementary Fig. 2g). The fact that both WT and NQO1-Tg mice have similar reduction in RER flexibility when fed HFD can be attributed to the source of energy consumed and reflects diet composition (predominantly fat) rather than differences in NQO1 levels or degrees of impaired glucose tolerance^32^. Significant reductions in VO_2_ and VCO_2_ levels were observed in WT mice on HFD vs. SD, whereas a much weaker impact of the diet emerged in NQO1-Tg mice (Supplementary Fig. 2h). Consumption of HFD was associated with higher heat production in mice of both genotypes (Supplementary Fig. 2h). Together, these results reveal that NQO1 transgenic mice exhibit significant improvements in glucose homeostasis and insulin sensitivity without obvious changes in body composition, food intake, and overall metabolic flexibility compared to WT littermates when fed HFD.

HFD elicits liver damage related with lipid accumulation and local inflammation. Histochemical analyses were carried out on fixed liver tissues of mice on HFD diet for 16 weeks to determine the effects of NQO1 overexpression on diet-induced increase in hepatic steatosis. Compared to WT, the liver of Tg mice weighed significantly less and were reddish-brown in color, exhibiting lower levels of fat infiltration and greater glycogen deposition (Fig. 2h). Lipid accumulation in the form of diglycerides and triglycerides was also significantly reduced in Tg vs. WT liver, approaching the levels seen in SD-fed mice (Fig. 2i).

To assess the impact of genetic overexpression of NQO1 at conferring protection from diet-induced insulin resistance, we used a fasting/feeding paradigm in which mice previously on either SD or HFD for 16 weeks were fasted for 24 h followed by a 3-h refeeding period with their respective diet (Fig. 3a, upper panel). Skeletal muscle extracts from SD-fed NQO1-Tg mice exhibited significant reduction in the levels of IR β-sub and activated phosphorylated form of Akt substrate of 160 kDa (AS160), a key player in insulin-mediated glucose uptake, while IRS-1 levels trended lower compared to muscles from WT littermates (Fig. 3a and Supplementary Fig. 2j). Conversely, muscle extracts from NQO1-Tg mice on HFD showed increased expression of IR β-sub and phospho-active AS160 (Fig. 3a). These changes were consistent with GIR profiles observed during the hyperinsulinemic–euglycemic glucose clamps, whereby diet (SD vs. HFD) either reduced or increased GIR in Tg mice vs. WT littermates (Fig. 2g).

HFD also triggered macrophage accumulation in adipose tissue, a condition favoring the production and secretion of pro-inflammatory adipokines and cytokines^33^. Immunohistochemical antigen labeling followed by H&E counterstaining was carried out to ascertain the impact of NQO1 overexpression on the percentage of CD68^+^ cells, a marker for total macrophage content, and on the overall adipose tissue structure and integrity. Despite similar expansion of fat depots in mice of both genotypes in response to HFD, H&E staining of eWAT from WT mice revealed the presence of cells in the interstitial space surrounding adipocytes, coincident with positive CD68^+^ staining (Fig. 3b). The number of CD68^+^ cells was significantly decreased in NQO1-Tg vs. WT animals (Fig. 3c). The gene expression levels of a panel of cytokines and adipokines was analyzed in eWAT by qPCR and the results of this analysis showed significant increases in TNFα and IL-1Rα mRNA levels in WT mice on HFD while the response of Tg animals was largely refractory to HFD feeding (Fig. 3d and Supplementary Fig. 2i). To further explore the molecular changes associated with improved insulin sensitivity in eWAT, extracts from the fasting/feeding experiment depicted in Fig. 3a were immunoblotted for IR β-sub and lipogenic enzymes (Fig. 3e, f). Significant reduction in the abundance of IR β-sub. was observed in eWAT from WT, but not Tg mice on HFD, whereas NQO1 transgenesis was associated with increased expression of several lipogenic enzymes in response to HFD, including acetyl-CoA carboxylase (ACC), ATP-citrate lyase (ACLY), and fatty acid synthase (FASN) with a trend toward higher lipin 1 levels (Fig. 3e, f).

Altogether, the data indicate that, although NQO1-Tg mice on HFD develop obesity, they remain insulin sensitive while being protected from liver steatosis. In addition, the lowering in the augmented inflammatory profile in eWAT by NQO1 transgenesis was accompanied by an accumulation of lipogenic enzymes in eWAT of HFD-fed mice.

### Translational control by NQO1

**Fig. 4:**
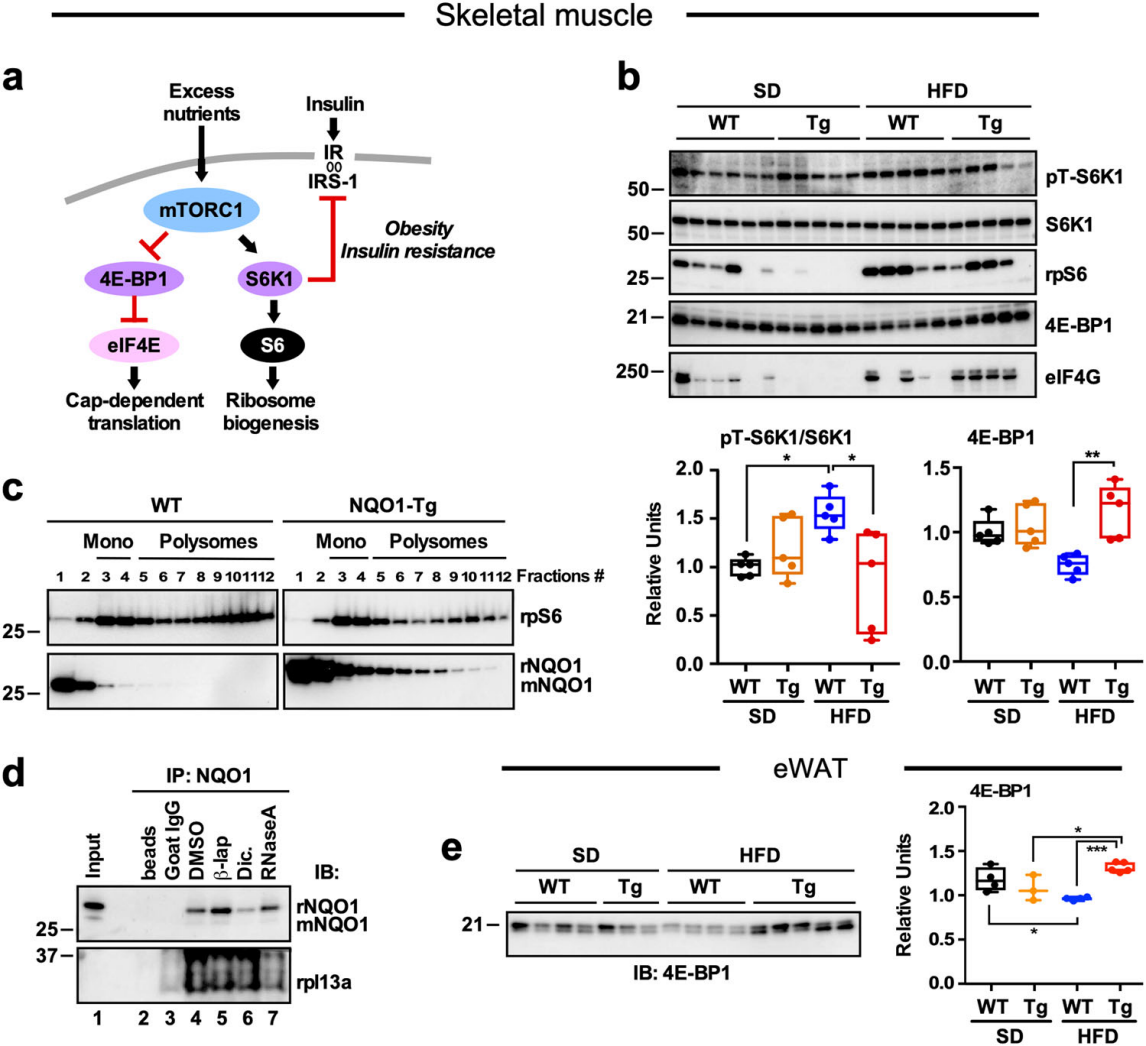
Effect of NQO1 transgenesis on downstream effectors of mTORC1 and translation machinery. **a** Diagram depicting the impact of excess nutrients on mTORC1 pathway activation and associated inhibition of insulin signaling. **b** *Top panel*, Skeletal muscle extracts from Fig. 3a were rerun and probed with antibodies against key downstream effectors of mTORC1, namely the phosphorylated and total forms of S6K1, ribosomal protein S6 (rpS6), 4E-BP-1, and eIF4G protein. *Bottom panel*, densitometric quantification of the phospho-active/total ratio of S6K1 and of 4E-BP1. Values are represented as boxplots with individual values (*n* = 5 per group). **c** Sucrose gradient fractionation of pooled cytosolic quadricep extracts from WT and NQO1-Tg mice on HFD. Fractions were resolved by SDS-PAGE and immunoblotted for rpS6 and NQO1 proteins. Monosomes are found in fractions 3–4, and polysomes in fractions 5–12. **d** NQO1:protein complexes in skeletal muscle homogenates were immunoprecipitated with goat anti-NQO1 antibody. Immunoprecipitates were incubated with DMSO, 10 μM beta-lapachone (β-lap.), 100 μM dicoumarol (Dic.), or 1 μg RNase A for 3 h at 4 °C, followed by immunoblot analysis (IB) with antibodies against rpl13a and NQO1. Control immunoprecipitations were carried out with magnetic beads alone and beads conjugated with goat IgG control. **e** *Left panel*, eWAT extracts from Fig. 3e were rerun and probed with anti-4E-BP1 antibody. *Right panel*, densitometric quantification. *, **, ***p < 0.05, 0.01, 0.001.

Tissues of individuals and mice considered obese exhibit increased activation of mTOR complex 1 (mTORC1) and insulin resistance^34,35^ (Fig. 4a). Previous studies have shown that reducing mTORC1 results in protection against the development of T2D and onset of diabetic nephropathy in mice^36–39^. Double deletion of the mTORC1 downstream targets and translational repressors, 4E-BP1 and 4E-BP2, in mice contributes to insulin resistance, increased S6K activity, and impaired Akt signaling in muscle, liver, and adipose tissue^40^. Conversely, increased 4E-BP-1 expression protects against HFD-induced insulin resistance in transgenic mice^41^.

To ascertain whether NQO1 transgenesis impacts skeletal muscle anabolic response via mTORC1 modulation, we reprobed skeletal muscle extracts from the fasting/refed experiment (Fig. 3a) with antibodies raised against downstream effectors of mTORC1, namely p70S6 kinase 1 (S6K1), ribosomal protein S6 (rpS6), and 4E-BP1 and eukaryotic translation initiation factor 4G (eIF4G). Phosphorylation of S6K1 modulates ribosomal protein synthesis and is important for regulating the balance between protein anabolic/catabolic processes^35^. Here, we found that the phosphorylated/total ratio for S6K1 increased significantly in HFD-fed WT mice (Fig. 4b), supporting the role of active S6K1 in promoting insulin resistance^39^ (Fig. 4a). In contrast, muscles of Tg mice on HFD had significantly lower levels of active S6K1 (as judged by phosphorylated/total ratio) and increased accumulation of 4E-BP1 in addition to maintaining normal levels of rpS6 and eIF4G proteins compared to WT muscles (Fig. 4b and Supplementary Fig. 2j). Of note, the levels of rpS6 and eIF4G proteins trended lower in muscles of NQO1-Tg mice vs. WT littermates fed SD (Fig. 4b and Supplementary Fig. 2j).

Because of the role of NQO1 in the regulation of mRNA translation and interaction with RNA-binding proteins^22,42^, we first performed polysome analysis by pooling cytosolic extracts from muscle tissues of WT vs. Tg mice on HFD and subjecting them to sucrose density gradient centrifugation (Fig. 4c). This method allows the separation of mRNAs into non-translating and actively translating fractions depending on their association with monosomes and polysomes, respectively^43^. Consistent with the role of ribosomes in protein synthesis, rpS6, a component of the 40S ribosome, can be found in polysomes of both WT and Tg muscles, along with NQO1 obtained from Tg muscle lysates (Fig. 4c). Association of NQO1 with polysomes was also observed in Tg mice on SD (data not shown). Thus, muscle homogenates from Tg animals overexpressing NQO1 exhibited higher levels of NQO1 associated with polysomes compared to WT samples. Next, a co-immunoprecipitation approach was used to examine a potential interaction between NQO1 and ribosomes in muscle cytosolic extracts. Immunoblotting anti-NQO1 immunoprecipitates was performed with antibodies to the ribosomal subunit rpl13a and the interaction of the two proteins was found (Fig. 4d). Immunoprecipitation using goat anti-NQO1 antibody was carried out either in the presence of the NQO1 activator, beta-lapachone (β-lap), the inhibitor dicoumarol, or RNase A treatment. It was found that treatment with β-lap and dicoumarol, two compounds reported to induce conformational changes within NQO1, resulted in differences in the recovery of NQO1 protein bands, which could be explained by differential exposure of the epitope to the antibody^44^. Nevertheless, immunoblot analysis confirmed the presence of rpl13a (Fig. 4d). These data also demonstrated that RNase A-treated NQO1 immunoprecipitates had marked reduction in the levels of rpl13a when compared with the dimethyl sulfoxide (DMSO)-treated sample, providing evidence that NQO1-RNA interaction mediates association with the translational machinery.

Similar to muscle, there was significant increase in the levels of 4E-BP1 in eWAT of HFD-fed Tg mice, consistent with NQO1 acting as a translational inhibitor across two main tissues responsible for metabolic regulation (Fig. 4e).

Altogether, these results indicate that NQO1 transgenesis confers protection from HFD-mediated alteration of whole-body glucose and lipid metabolism through mechanisms that likely include translational control.

### Multi-organ and serum metabolomics in WT and Tg mice subjected to SD and HFD

**Fig. 5:**
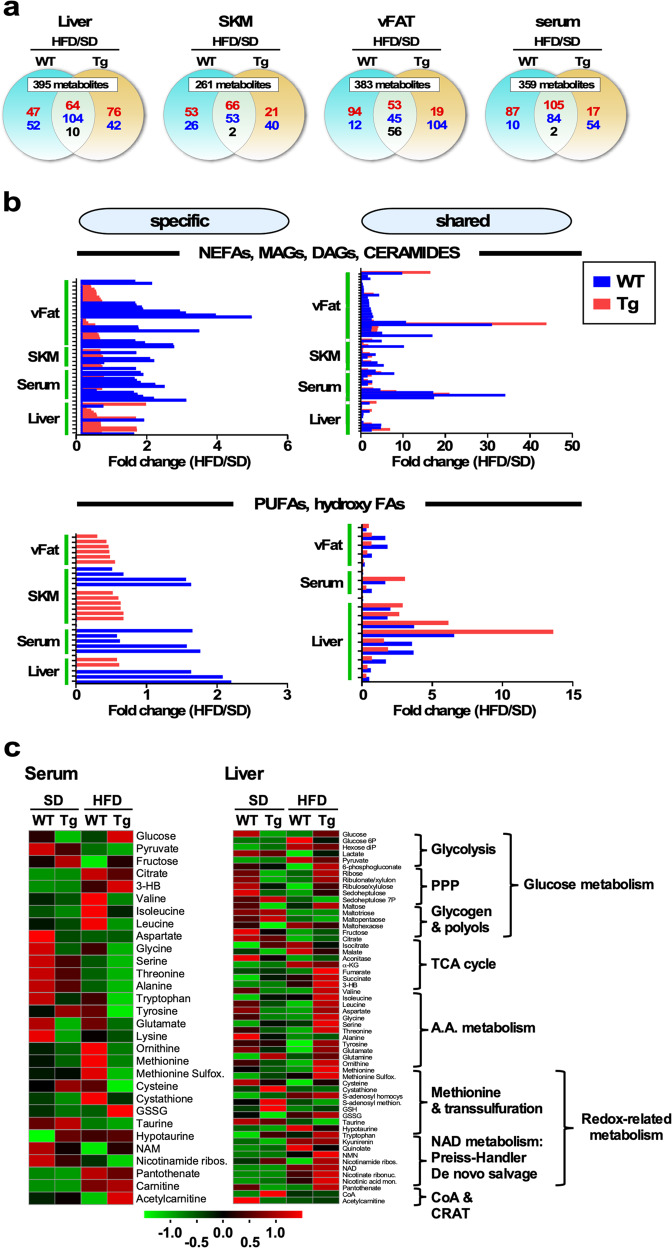
Untargeted metabolomics analysis on the effect of global NQO1 overexpression in several tissues and serum of WT and NQO1-Tg mice-fed SD or HFD. **a** Two-way Venn-diagrams depicting the distribution of unique and common metabolites in WT and NQO1-Tg mice-fed HFD vs. SD. Untargeted metabolomics analysis was carried out in liver, skeletal muscle (SKM), visceral WAT (vFAT) and serum. Upregulation (red font), downregulation (blue font), and reciprocal regulation (black font) of significantly impacted metabolites (defined as fold-change >1.5 or <0.67) are depicted. **b** Distribution of various classes of lipids (e.g., NEFAs, MAGs, DAGs, ceramides, PUFAs, hydroxy FAs) in the indicated tissues and serum of WT and NQO1-Tg mice-fed HFD vs. SD. **c** Heatmap visualization of metabolites in serum (left) and liver (right) of WT and NQO1-Tg mice-fed SD or HFD. Upregulation (red font), downregulation (green font).

To investigate the effect of NQO1 overexpression and diet on organismal metabolism, untargeted mass spectrometry metabolomics was performed in serum and tissues (liver, skeletal muscle and visceral adipose tissue) of mice using a multiplatform approach (Fig. 5 and Supplementary Figs. 3 and 4).

Tissues were harvested from mice that underwent the fasting/feeding paradigm described in Fig. 3a. Metabolites profiling using untargeted metabolomics revealed 766 metabolites in liver (SD/HFD: *n* = 5/5); 751 metabolites in serum (SD/HFD: *n* = 5/5); 655 metabolites in skeletal muscle (SD/HFD: *n* = 5/5) and 632 metabolites in visceral fat (SD/HFD: *n* = 5/5). Separated by tissue and serum, metabolite profiles were analyzed as a function of diet by Partial Least Square Discriminant Analysis (PLS-DA), a cross-validated multivariate supervised clustering/classification method from MetaboAnalyst 4.0^45,46^. Both genotypes exhibited a clear separation between the two diets (Supplementary Fig. 3a).

Since the effects of NQO1 overexpression on physiological outcomes were more advantageous in HFD, we decided to analyze, in each tissue and serum, the ratio HFD/SD of metabolites with fold-changes ≥1.5 and ≤0.67 to dissect specific and shared metabolites for WT and Tg. Utilizing Venn-diagrams, we sought to find out which metabolites/pathways were specifically and more significantly influenced by genotype and, among the shared metabolites, which genotype had the greater effect (Fig. 5a). Supplementary Table 1 describes the “shared” and “specific” metabolites above or below the abovementioned fold-change threshold.

Monitoring changes in amino acid metabolism may help to understand the effects of NQO1 transgenesis and diet on muscle insulin sensitivity. As depicted in Supplementary Fig. 4, marked increase in Ile, Leu, and Met was observed in skeletal muscle and visceral fat of HFD-fed WT mice. Given the role of these amino acids on mTORC1 activation and glucose homeostasis^47,48^, these findings could, in part, explain the decreased S6K1 activation observed in Tg mice on HFD (Fig. 4b) and support mechanism of protection against diet-induced insulin resistance mediated by mTORC1/S6K1/IRS-1 signaling (Fig. 4a)^39^. Moreover, *S*-adenosyl methionine levels were significantly reduced in skeletal muscle of Tg mice and this could also help to clarify the effect on mTORC1 activation in light of recent findings on SAMTOR as an *S*-adenosyl methionine sensor for the mTORC1 pathway^49^.

As ~50% of the detected metabolome is constituted by metabolites of lipidic nature (and is roughly maintained within “shared” and “specific” groups), we asked whether NQO1 was affecting lipid metabolism. We focused on the differences in each tissue/serum between WT and Tg in eight main lipid classes including non-esterified fatty acids (NEFAs), monoacylglycerols (MAGs), diacylglycerol (DAGs), polyunsaturated fatty acids (PUFAs), hydroxylated fatty acids, ceramides, and acylcarnitines. A glanced view of lipid class distribution specific for each genotype or shared between WT and Tg is depicted in Supplementary Fig. 3b. With a few exceptions, and regardless of the diet, NEFAs, MAGs, DAGs and ceramides were more elevated in WT than in Tg, while PUFAs, but not hydroxy-fatty acids, were more elevated in the Tg (Fig. 5b and Supplementary Fig. 3c). For reasons of clarity, the lipid species changed between genotypes are presented in Supplementary Table 2.

The enhanced abundance of acylcarnitines in skeletal muscle and liver from HFD-fed Tg vs. WT mice suggested buffering of the mitochondrial pool of acetyl-CoA (Supplementary Fig. 3c). Carnitine *O*-acetyltransferase (CRAT) is an enzyme that catalyzes the conversion of short-chain acyl-CoAs to their membrane permeant acylcarnitine derivatives. It follows that CRAT down-modulates mitochondrial acetyl-CoA and avoids protein hyperacetylation.

Altogether, the data obtained show an apparent overall positive effect of NQO1 overexpression on lipid metabolism in HFD-fed mice, likely due to better handling and decreased abundance of lipotoxic species along with increased levels of beneficial bioactive lipids, suggesting that signaling processes starting at the plasma membrane, e.g., by PUFAs, could be mediating salutary actions on inflammatory and immunity-related processes^50,51^. Moreover, the higher levels of acylcarnitines exhibited by muscle and liver tissue from Tg vs. WT agrees with improved management of lipid excess by hindering the inhibitory effect of mitochondrial protein hyperacetylation on metabolic fluxes^52^.

### Diet- and genotype-dependent increase in protein acetylation

We assessed protein acetylation in skeletal muscle to further investigate the status of this posttranslational modification in the same 10-mo-old mice that underwent the fasting/refeeding paradigm depicted in Fig. 3a.

**Fig. 6:**
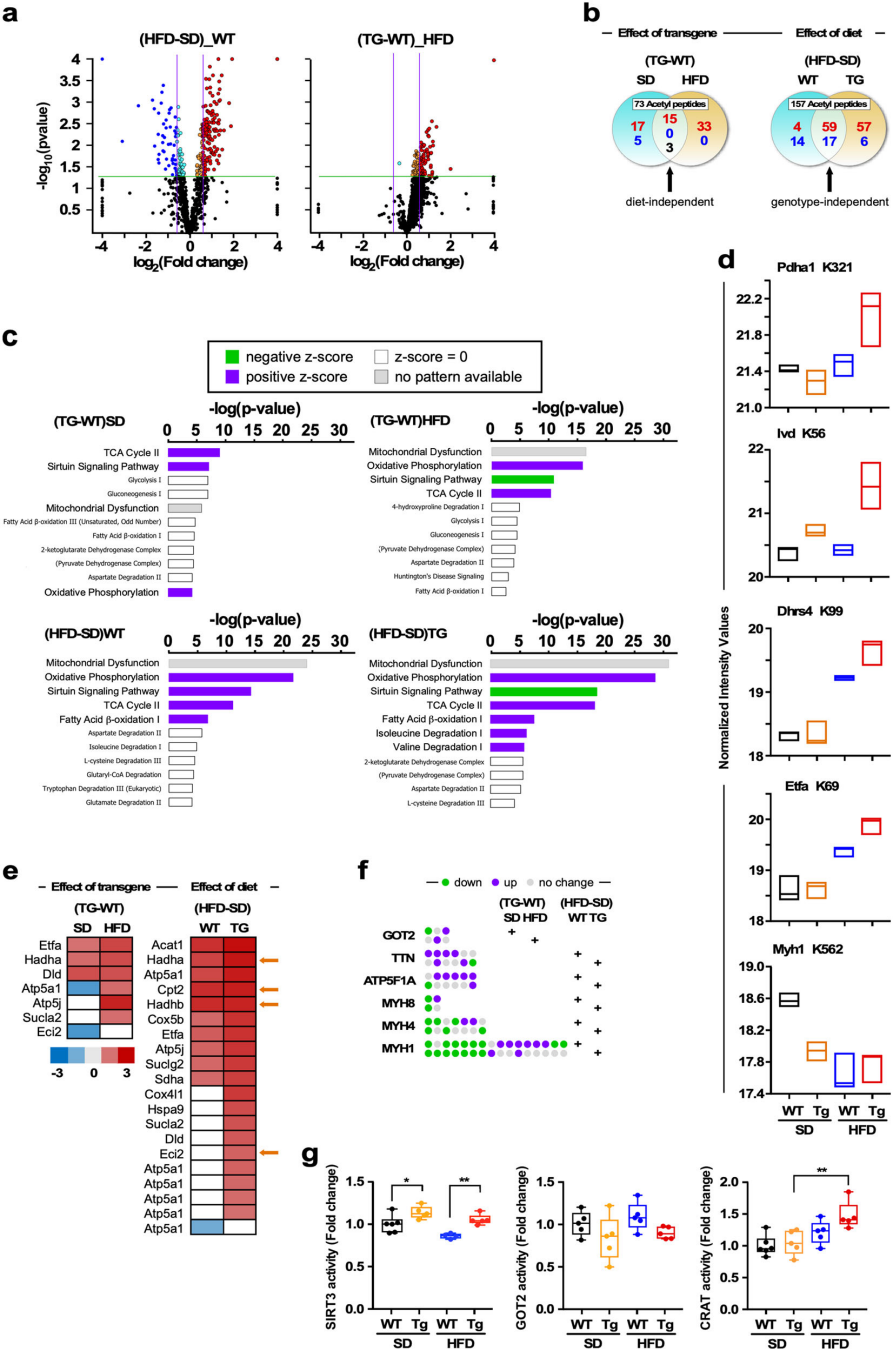
Effect of global NQO1 overexpression and diet composition on the lysine acetylome in skeletal muscle and on enzymatic activities impacting acetyl-lysine turnover in liver extracts. **a** Volcano plots of the lysine acetylome in skeletal muscle performed in the WT and NQO1-Tg mice fed either SD or HFD for 16 weeks. Graphs show the log2 fold-change of lysine acetylation between the indicated experimental groups of mice. *n* = 3 mice per group. **b** Venn-diagrams depicting the distribution of lysine-acetylated proteins in skeletal muscle of WT and NQO1-Tg mice on SD or HFD diet in response to the “TG-WT” and “HFD-SD” pairwise comparisons. Shared elements constitute attributes that are either diet- or genotype-independent, as indicated. Note that the digestion of a few proteins (e.g., myosin 1 and myosin 4) generated several peptides with the same acetylation site without and with other modifications such as peptide deamination and methylation. For this reason, these “shared” peptides were counted as one. Red font, hyperacetylation; blue font, hypoacetylation; black font, acetylation going in opposite direction. **c** Top pathways calculated by Ingenuity Pathway Analysis (IPA) using enrichment p-values. Bias-corrected *z*-score [positive (purple box), negative (green box), non-significant (white box)] are depicted as well as pathways without available pattern (gray box). **d** Normalized intensity values for a select group of acetyl peptides differentially influenced by diet x genotype interaction are depicted as boxplots (*n* = 3 per group). The lysine modification sites are shown. These data were analyzed using a two-way ANOVA. See Table S4 for complete list of acetyl peptides. **e** Heatmaps visualization of a select group of acetylated proteins in skeletal muscle that are known targets of the SIRT3 deacetylase. Left panel, effect of NQO1 overexpression; right panel, effect of diet. Upregulation (red), downregulation (blue). **f** Visualization of six proteins containing multiple lysine acetylation sites whose profiles are altered by genotype or diet in the indicated pairwise comparisons. **g** SIRT3, GOT2 and CRAT activities were performed in liver extracts. The data were analyzed using a two-way ANOVA. *p < 0.05; **p < 0.01.

In general, nutrient excess and NQO1 transgenesis were associated with increased protein acetylation in skeletal muscle but NQO1 reduced the extent of overall hyperacetylation induced by HFD (Fig. 6a). A total of 73 and 157 unique peptides with significant pairwise differences in average fold-change in lysine acetylation between Tg:WT and HFD:SD, respectively, were detected, of which 18 and 76 were found to be unresponsive to diet and genotype (Fig. 6b). The complete list of acetylated proteins and their direction of change can be found in Supplementary Table 3. The potential functional relationships between acetylated proteins were then analyzed with STRING functional network association (http://string-db.org) (Supplementary Fig. 5 and Supplementary Table 4, and data not shown). Using a high confidence cutoff of 0.7, a strong functional connectivity between 8 to 65 protein interactors was found among various pairwise comparisons. Most of the lysine acetylation-associated networks demonstrated a very high enrichment probability (i.e., *p* < 10e^−16^), except for WT mice on HFD, which showed a protein–protein interaction (PPI) value of only 0.0102 (Supplementary Table 4). Mitochondrial bioenergetics, cellular oxidation-reduction, and drug and nucleotide metabolism were among the top highly enriched biological processes mapped from Gene Ontology database (Supplementary Table 5). Similar results were obtained when the same dataset of acetylated proteins in NQO1-Tg mice under HFD was analyzed with the Ingenuity Pathway Analysis platform that identified oxidative phosphorylation, TCA cycle, and fatty acid β-oxidation enhancement (Fig. 6c).

Differences in lysine acetylation between experimental groups were considered significant at fold-change ≥1.5 in either direction, *p* < 0.05, and false discovery rate <1.2%. Two-way analysis of variance (ANOVA) detected 44 acetyl peptides with combined diet-genotype interaction underscoring a higher impact of NQO1 transgenesis on muscle lysine acetylome from HFD-fed mice (Fig. 6d and Supplementary Table 6). Interestingly, we found that NQO1 overexpression exacerbated the acetylation levels of a select group of SIRT3 targets in the skeletal muscle of HFD-fed mice, including the mitochondrial β-oxidation enzymes Hadha, Hadhb, Cpt2, and Eci2 (Fig. 6e, orange arrows). From the muscle acetylome, only six proteins were found to contain several acetylation sites, including the mitochondrial aspartate aminotransferase (GOT2) and ATP synthase F1 subunit A (ATP5F1A), titin, and the contractile proteins myosin 1, 4, and 8 (Fig. 6f). Diet and NQO1 transgenesis differentially influenced the direction and location of the acetylated lysine residues within each of these proteins (Fig. 6f), likely affecting skeletal muscle metabolism and contractility. On the other hand, myosin 1 and 4 were among the few proteins that were acetylated at baseline but experienced deacetylation with HFD regardless of the genotype (Fig. 6f).

Altogether, these data show that HFD induces robust acetylomic changes in the muscle, which are distinctly different when comparing WT and NQO1-Tg animals (Fig. 6a). Clearly, NQO1 overexpression reduces global protein hyperacetylation induced by HFD, suggesting an overall improvement in metabolic phenotype. When combined with our metabolomic analysis, it appears as though AcCoA buffering via acylcarnitines may play a role in these altered acetylomic profiles. These results are consistent with the hypothesis that activation of metabolic mechanisms counteracting potential damage by lipid overabundance were favored by NQO1 overexpression.

Even though each organ differs in its metabolic and proteomic profile, there was not enough skeletal muscle tissue available to measure SIRT3, GOT2, and CRAT enzymatic activities in vitro. This led us to measure their activities in liver extracts under the four experimental conditions, as liver helps maintain organismal metabolic homeostasis. NQO1 transgenesis significantly increased hepatic SIRT3 activity regardless of the diet whereas an upregulation in CRAT activity was observed in NQO1-Tg mice on HFD compared to SD-fed animals (Fig. 6g). Neither hepatic GOT2 activity nor the levels of SIRT3, GOT2 and CRAT proteins were impacted under these experimental conditions (data not shown).

### NQO1 improves glucose and redox metabolism in different organs from HFD-fed Tg mice

Next, we asked whether the apparent lower dependence of Tg vs. WT mice on lipids entails an increase in glucose and redox metabolism. To address this, we performed an untargeted analysis of metabolites from glucose- and redox-related metabolic pathways in tissues and serum.

Average heat maps of glucose- and redox-related metabolomes from WT and Tg mice subjected to SD or HFD are displayed in Fig. 5c and Supplementary Fig. 4. Amino acids were also included in the analysis because the liver plays a key role in endogenous glucose production through gluconeogenesis^53^. Remarkably, compared to WT, livers of HFD-fed Tg mice exhibited enhanced glucose and redox metabolism (Fig. 5c, see metabolites’ relative abundance from glycolysis, glycogen synthesis, pentose phosphate, TCA cycle, and the NAD salvage pathways), even though NQO1 expression was not significantly changed. Unlike in WT animals, the three independent pathways that maintain NAD^+^ levels, i.e., Preiss-Handler, *de novo*, and salvage pathways^54^, appear to be expressed in liver from HFD-fed Tg mice (Fig. 5c), as evidenced by the presence of several intermediates from NAD^+^ metabolism, such as tryptophan, kynurenine, quinolate, nicotinamide mononucleotide (NMN), nicotinamide riboside, NAD, nicotinate ribonucleoside, and nicotinic acid mononucleotide.

**Fig. 7:**
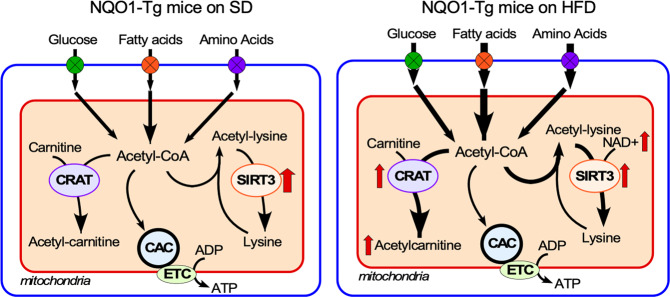
Working model illustrating cooperative regulation of carnitine acetyltransferase (CRAT) activity coupled with SIRT3-mediated removal of the excess mitochondrial protein acetylation in the liver of NQO1-Tg mice-fed high-fat diet (HFD). NQO1 transgenesis increases the availability of NAD^+^, an essential cofactor for SIRT3 activity.

The abundance of several amino acids in the liver increased concomitantly with their depletion in serum and enhancement of glucose in the circulation (Fig. 5c). In response to HFD, the liver from NQO1-Tg mice appears to be acting as a sink of amino acids from serum, accompanied by their degradation and subsequent replenishment of the TCA cycle via anaplerosis (Figs. 5c and 7).

Pathway analysis indicated significant activation of CoA biosynthesis in Tg liver in agreement with the higher levels of pantothenate in Tg vs. WT liver. In addition, lower levels of acetylcarnitine and CoA suggest better handling of lipid oxidation and less dependence on fat metabolism.

The pattern of glucose metabolism activation appears to be more specific in skeletal muscle than in liver, consisting of more glycogen synthesis, whereas visceral adipose tissue exhibited a more pronounced and consistent glucose metabolism as can be judged from metabolites abundance. Redox metabolism, including NAD^+^ and glutathione synthesis, was active in both organs (Supplementary Fig. 4).

Altogether, the data obtained are consistent with the interpretation that under substrate excess and oxidative conditions promoted by HFD, NQO1 overexpression improves whole-body glucose metabolism and insulin sensitivity, which might be due to less dependence on lipid metabolism. The observed improvement in NAD^+^ metabolism is compatible with greater protection from hepatic steatosis and macrophage infiltration of fat depots by global overexpression of NQO1.

## Discussion

NQO1 confers protection against metabolic disorders that are associated with chronic inflammation and insulin resistance. To provide new insights into the beneficial role of NQO1 and its upstream transcriptional regulator Nrf2 in metabolic processes and glucose homeostasis, we used genetic mouse models that promoted either an inducible Nrf2 upregulation across most tissues (*Keap1*-KO) or whole-body overexpression of rat NQO1 (NQO1-Tg). The major results of this work show that NQO1 transgenic mice under HFD exhibit significant improvements in glucose homeostasis and insulin sensitivity without obvious changes in body composition, food intake, and overall metabolic flexibility. In *Keap1-*KO mice, the NQO1-elicited enhancement of whole-body glucose metabolism appears to be mediated by the constitutive activation of Nrf2, a pleiotropic regulator of the oxidative stress response. The major difference between *Keap1*-KO and NQO1-Tg mice in terms of the molecular mechanisms of regulating intermediary metabolism stems is that deletion of Keap-1 potentially affects the expression of >200 genes downstream of Nrf2 with conflicting outcomes in terms of glucose or lipid homeostasis^2,55,56^, whereas selective overexpression of NQO1 protects mice from diet-induced insulin resistance. While genetic deletion of NQO1 decreases glucose and fatty acid metabolism^19^, we show that global NQO1 transgenesis elicits changes in the level of lipid metabolites including PUFAs.

The mechanism proposed is that NQO1 overexpression in skeletal muscle and WAT improves insulin sensitivity owing to attenuation of mTORC1 signaling via S6K1 and elevation of 4E-BP1 levels. These events have been independently linked to preservation of insulin sensitivity and amelioration of lipid profile under metabolic stress condition^39–41^, and we speculate that NQO1 association with translating ribosomes may play a role in regulation of metabolic enzymes via mechanisms involving control of mRNA translation. We and others^42^ have previously reported that NQO1 is an RNA-binding protein and this function could mediate interaction with the translation machinery and regulate expression of several mRNAs. Further molecular details will be investigated in future studies, but the molecular changes described herein are consistent with the hypothesis that under HFD, attenuation of pathways downstream of mTORC1 improves both glucose and lipid homeostasis, helps to preserve insulin sensitivity, and results in reduced liver steatosis and improved muscle function. Of note, many of these aspects were observed in two independent models (Keap-1 KO and NQO1-Tg mice) that shared NQO1 overexpression in organs crucially involved in the maintenance of energy homeostasis.

Improvement of redox metabolism, as can be judged by changes in the metabolome exhibiting a consistent pattern of enhancement associated with NAD^+^ metabolism and pentose phosphate pathways (Fig. 5c), agrees with observations showing that NQO1-Tg mice maintain insulin sensitivity while being protected from liver steatosis and macrophage infiltration into fat depots. In addition to conferring protection against HFD-mediated alteration in glucose metabolism, the NQO1 transgenesis also imparts improvement of lipid metabolism, as indicated by decreased abundance of lipotoxic species (e.g., ceramide, MAGs, DAGs) (Fig. 5b) and increased bioavailability of beneficial lipids (Supplementary Fig. 3c) such as PUFAs, namely, eicosapentanoic and docosahexanoic acids, which are known to have salutary actions on inflammatory and immunity-related processes^50,51^. These findings are consistent with the known activity of NQO1 as an antioxidant and protective enzyme against lipid peroxidation^57^. In the same vein, the higher levels of acylcarnitines exhibited by muscle and liver tissue from NQO1-Tg vs. WT mice (Supplementary Fig. 3c) are consistent with improved management of lipid excess and dampening the acetylation of mitochondrial proteins, a general inhibitory marker of metabolic fluxes^52^. Lysine acetylome pathway analysis suggested the modulation of proteins related to intermediary metabolism in HFD-fed Tg mice. Overall, the data available shows that NQO1 overexpression favors the activation of metabolic mechanisms that counteract the damaging effects of lipid oversupply.

In addition to providing antioxidant activity via scavenging superoxide and promoting antioxidant forms of ubiquinone and vitamin E^58^, NQO1 prevents the degradation of the coactivator PGC-1α^26^. PGC-1α contributes to lipid turnover and links fatty acid import across cellular membranes to mitochondrial oxidative pathways, including β-oxidation^59,60^. Changes in cellular NADH/NAD^+^ ratio caused by redox events have recently been reported to alter NQO1 conformation and its ability to bind to or dissociate from target macromolecules^44^. NQO1 supports sirtuin function and its role in transcription of target genes and sensing of mitochondrial stress. We surmise that the induction of NQO1 stabilizes PGC-1α levels and promotes the deacetylation of target substrate proteins via generation of NAD^+^ and/or interaction with SIRTs, which in turn help reduce oxidative damage to proteins and decrease the levels of inflammatory cytokines that are associated with HFD consumption. Therefore, NQO1, sirtuins, and PGC-1α may act as a metabolic rheostat in the control of mitochondrial function and fatty acid oxidation by sensing the NADH/NAD^+^ ratio.

Induction of Nrf2 in young adult *Keap1*-KO mice slowed their weight gain trajectory and conferred protection against diet-induced obesity while maintaining diurnal metabolic flexibility in their use of fuels for energy needs. Improved glucose homeostasis was observed in *Keap1*-KO mice fed an obesogenic diet when subjected to glucose but not to insulin tolerance test, consistent with an enteroendocrine-sensing mechanism implicating intestinal K cells-mediated release of incretins and related polypeptides^61,62^. Selective induction of endogenous NQO1 was seen in metabolically relevant target tissues of *Keap1*-KO mice including skeletal muscle, liver, and WAT while expression of other Nrf2 targets such as the antioxidants GCLC, HO-1 and SOD2 was minimally impacted. These results led us to consider the possibility that NQO1 induction may have contributed to some of the beneficial metabolic effects observed in *Keap1*-KO mice. Indeed, global NQO1 transgenesis was accompanied by whole-body protection of HFD-fed mice against metabolic dysfunction, presumably due, in large part, to the marked expression of the transgene in skeletal muscle. Moreover, WAT also expressed the transgene together with sharp reduction of macrophage infiltration, a readout of diet-induced low-grade inflammation. It is well accepted that the consumption of HFD promotes the storage of fatty acids in the liver instead of being delivered to WAT, resulting in liver enlargement caused by the development of steatosis and dysregulation of hepatic immune function^63,64^. Our results indicate the protection of liver size and morphology in NQO1-Tg mice on HFD despite the lack of NQO1 overexpression in this organ. Metabolomics analysis revealed the release of different lipid classes in the serum (e.g., PUFAs, hydroxy-fatty acids) by the adipose tissue that could, in principle, be better processed and fully oxidized in the liver, which in turn reduces steatosis and hepatic inflammation in NQO1-Tg mice (Supplementary Fig. 3c). These data are consistent with the “central role of adipose tissue in regulating systemic glucose homeostasis through the control of liver and skeletal muscle metabolism”^65^.

Lipid oversupply can also elicit extensive protein acetylation, as suggested by the activation of carnitine acetyltransferase (CRAT) to buffer excess of the acetyl donor acetyl-CoA, in agreement with the increase in acylcarnitines. Further investigation of the lysine acetylome unveiled high connectivity among acetylated proteins, mainly involved in pathways of mitochondrial metabolism such as oxidative phosphorylation, TCA cycle, and β-oxidation. These findings suggested that the lysine acetylome could constitute a readout of a widespread metabolic coordination affecting mitochondrial energetics. CRAT deficiency has been shown to increase acetyl-CoA levels rendering mitochondrial matrix proteins more prone to diet-induced lysine acetylation and enhanced fat oxidation without overt impairment in mitochondrial respiration. Our metabolomics data suggest that under HFD, NQO1-Tg mice might be able to better control acetyl-CoA concentration, possibly through CRAT, as the increased levels of acylcarnitine derivatives in muscle and liver show, thus moderating acetylation-mediated inhibition of metabolic fluxes during HFD-induced stress. In addition of catalyzing the reversible transfer of acyl groups from carnitine to CoA, CRAT has an important role in the mitochondrial transport of FA for β-oxidation. Our results show that consumption of HFD in NQO1-Tg mice was associated with significant increase in CRAT activity in the liver, consistent with the accumulation of acetylcarnitine. Figure 7 depicts our interpretation of the metabolome and acetylome data.

It has been recently shown that a double knockout of CRAT and SIRT3 in mice subjected to HFD produces mitochondrial protein hyperacetylation that did not affect mitochondrial respiration per se but elevated ROS release^66^. Further assessment of the individual knockouts of CRAT or SIRT3 led these authors to suggest that SIRT3-mediated acetyl-lysine turnover rather than acetyl-lysine stoichiometry modulates substrate utilization and glucose homeostasis in skeletal muscle^66^. In agreement with these findings, we found that NQO1 overexpression under HFD favors an increase in the activities of CRAT and SIRT3 that buffers mitochondrial acetyl group availability while counteracting mitochondrial protein hyperacetylation in the muscle concomitantly with enhancement of glucose and NAD metabolism in liver, skeletal muscle and adipose tissues.

Our data indicates that acetylation of enzymes from central catabolism, as they relate to mitochondrial energetics-redox functions, was increased in skeletal muscle of Tg vs. WT mice, both under SD and HFD (Supplementary Table 3), with the exception of mitochondrial F1F0 ATP synthase (ATP5A1, subunit a) in which acetylation was decreased ~2-fold in Tg under SD but increased under HFD by 1.6-fold. Mitochondrial malate dehydrogenase (MDH2) acetylation has been shown to be stimulatory, at least in humans, although inhibitory in mouse liver^52^. Acetylation represents, in most known cases, an inhibitory mark indicative of diminished metabolic fluxes. Apparently, a significant increase in mitochondrial protein acetylation levels affected a subset of mitochondrial proteins that are targeted by SIRT3, among them enoyl-CoA hydratase/3-hydroxyacyl-CoA dehydrogenase (Hadh) from β-oxidation, the activity, which is stimulated by acetylation^52,67^ or ATP5F1A from oxidative phosphorylation, that is inhibited by acetylation^52,68^. The malate-aspartate shuttle transfers NADH from cytosol to mitochondria to support glycolysis-dependent ATP production. Interestingly, such changes in mitochondrial NADH/NAD^+^ redox are directly affected by glutamate oxaloacetate transaminase 2 (GOT2), a mitochondrial enzyme whose activity also contributes to tryptophan catabolism^69,70^ and amino acid metabolism^71^. Lastly, GOT2 (also known as fatty acid binding protein 1) has been found to facilitate cellular uptake of long-chain FFAs^72^, whose accumulation contributes to hepatic lipotoxicity. Our findings showing elimination of HFD-associated hepatic steatosis and reduction in triglyceride levels in NQO1-Tg livers are consistent with decreased GOT2 activity.

GOT2 activity is inhibited through SIRT3-mediated deacetylation^73^. Here, the increase in hepatic SIRT3 activity was associated with a trend toward lower activity of GOT2, an enzyme harboring multiple acetylation sites. Since the percentage of acetylation corresponding to the respective total abundance of a specific protein is unknown, we are unable at this point to ascertain the impact of acetylation on metabolic functions. However, we know that, compared to WT, HFD-fed NQO1-Tg mice exhibit enhanced activity of main pathways of glucose and NAD^+^ metabolism in liver, skeletal muscle, and adipose tissue of mice subjected to a 24-h fasting/3-h refeeding protocol. These observations are compatible with higher insulin sensitivity, improved whole-body glucose homeostasis, less oxidative stress, and higher sirtuin activity. Indeed, NQO1-Tg mice showed improved lipid metabolism management as revealed by relatively lower levels of lipotoxic species such as mono- and diacylglycerols and ceramide, accompanied by salutary long chain, unsaturated, hydroxy-fatty acids. These metabolomics findings are consistent with protection from liver steatosis and macrophage infiltration in fat depots in NQO1-Tg vs. WT mice, as expected from more efficient fat utilization, less dependence on fats to the expense of glucose metabolism, as well as lower oxidative stress, in turn compatible with less inflammation. Together, these findings support the idea that NQO1 overexpression confers protection by moderating acetylation of mitochondrial proteins in response to nutrient excess.

There are limitations to this study: The mechanisms linking modulation of translation by NQO1 and improvement of serum lipid profile, or other previously reported effects such as stabilization of PGC-1α are not clear at this point and warrant further investigation. Moreover, NQO1 may improve metabolism through functions in muscle or eWAT, but the differential roles of these tissues in this context are not delineated. Lastly, it is unclear how the effect of NQO1 on fatty acid β-oxidation stems from its impact on carnitine shuttling of long-chain fatty acids across the mitochondrial inner membrane. Nevertheless, plans are underway to assess the benefits of NQO1 in improving sirtuin-mediated epigenetic regulation of fatty acid metabolism in adipocytes.

NQO1 transgenesis results in enhanced glucose homeostasis, insulin sensitivity, and lipid oversupply management without changes in body composition, food intake, and overall metabolic flexibility. The NQO1-elicited enhancement of whole-body glucose metabolism appears to be mediated, at least in the *Keap1*-KO mouse model, by increases in the constitutive NRF2 transcriptional activity, a pleiotropic regulator of the oxidative stress response. Overexpression of NQO1 in skeletal muscle and eWAT from NQO1-Tg mice was accompanied by the inhibition of the anabolic response and protein translational activity of mTORC1 in both organs, which could ultimately result in stress reduction in the liver and improved hepatic function. The systemic nature of the response to NQO1 transgenesis is also revealed by the substantial change in the metabolism of skeletal muscle, liver, and the adipose tissue that involve different beneficial lipid classes. The data presented underscore some key components of hepatic nutrient-sensing pathways, particularly CRAT and SIRT3, in response to NQO1 transgenesis, which are responsible for attenuating diet-induced imbalance in glucose and lipid metabolism, cellular redox, oxidative stress, and mitochondrial dysfunction.

## Methods

### Genetic deletion of KEAP1 in mice, husbandry, and diets

Tamoxifen-inducible *CMVCre-Keap1*^*flox/flox*^ mice were generated by crossing *Keap1*^*flox/flox*^ mice with CAG-CreERT2^+^ mice (The Jackson Laboratory, Bar Harbor, ME) on a C57BL/6J background. After weaning, male *CMVCre-Keap1*^*flox/flox*^ mice and age-matched *Keap1*^*flox/flox*^ littermates were fed either standard rodent chow (kcal: 14% fat, 54% CHO, 32% protein; Harlan, Madison, WI) or were switched to a Western diet (WD; kcal: 40% fat, 43% CHO, 17% protein; cat # D12079B; Research Diets, Inc., New Brunswick, NJ); see Supplementary Table 7 for composition. By 10 weeks of age, whole-body Cre-mediated deletion of *Keap1* was induced by intraperitoneal (i.p.) injection of tamoxifen (TAM; Sigma) for 5 days (75 mg/kg BW). Deletion of Keap1 exons 2–3 was confirmed by PCR analysis using the following primers: forward 5′-CGA GGA AGC GTT TGC TTT AC-3′, reverse 5′-GAG TCA CCG TAA GCC TGG TC-3′. Mice were housed at 22–24 °C on a 12-h light/dark cycle (lights on at 6:00 AM) with free access to food and water. Body weight and food intake were measured weekly for 26 weeks. For tissue collection, mice were euthanized, tissues were surgically removed, immediately weighted and either fixed or frozen in liquid nitrogen to be stored at −80 °C. All animal experiments were performed under a protocol (#MO19H128) approved by the Johns Hopkins University Animal Care and Use Committee.

### NQO1 transgenesis, experimental validation, and dietary intervention

The rat NQO1 gene was cloned into the pRC/CMV-rDTD plasmid^74^. The transgene insert was cleaved from the DNA cloning vector by digestion with SwaI and NruI restriction enzymes. The purified transgene was microinjected into fertilized C57BL/6J eggs at the University of Michigan Transgenic Animal Model Core Facility (http://www.med.umich.edu/tamc/). The construct was stably incorporated into the genome, and expression of the gene was under the control of the human cytomegalovirus immediate-early promoter and the SV40 polyadenylation sequences.

Southern blotting was performed by Taconic (Germantown, NY). A ~360 bp probe was used for hybridization following plasmid digestion with restriction enzymes, MluI and SnaBI, to confirm the presence of the transgene. EcoRV was used to digest the mouse genomic DNA prior to hybridization.

For quantitative reverse transcription PCR (RT-PCR), total RNA was extracted from frozen tissue samples using the RNeasy kit (Qiagen, Valencia, CA). Complementary DNA was synthesized from total RNA with the High-Capacity cDNA reverse transcription kit (Applied Biosystems, Foster City, CA). The quantitative RT-PCR was performed on individual cDNAs by using SYBR® Green PCR master mix in StepOne plus Real-time PCR system (Applied Biosystems). The primer sequences for mouse NQO1 were: 5′-TTCTCTGGCCGATTCAGAG-3′ (forward) and 5′-GGCTGCTTGGAGCAAAATAG-3′ (reverse) and those for rat NQO1 were: 5′- GTGCTTGTAGCAGGATTCGC-3′ (forward) and 5′- GCAGAGAGTACATGGAGCCG-3′ (reverse). mRNA expression was calculated by the 2−ΔΔ*CT* method normalized to the expression of GAPDH.

NQO1-Tg mice and WT littermate controls were housed four per cage in a room maintained at a constant temperature (20°–22 °C) in a light:dark 12:12-h schedule according to animal protocols and NIH guidelines. For metabolic experiments, 6-mo-old male mice were placed on either SD (Harlan rodent diet 2018) or HFD (diet D12492, Research Diets, Inc., New Brunswick, NJ) ad libitum with free access to water. Body weight and food intake were measured every two weeks for a period of 10 weeks. In vivo experiments were performed in marked timepoints (weeks) after starting with diet (Fig. 2d).

### Body composition and indirect calorimetry

Body composition was assessed by quantitative NMR (Echo-MRI 100) to determine total body fat and lean mass, which were expressed as percent body mass. For indirect calorimetric analyses, heat production, locomotor activity, O_2_ consumption (VO_2_), CO_2_ production (VCO_2_), and RER (defined as VCO_2_/VO_2_) were monitored over 48 h after adaptation of the mice to individual metabolic cages and were assessed by using the Comprehensive Laboratory Animal Monitoring system (Columbus Instrument, Columbus, OH). Body composition and metabolic cage experimentation were analyzed by the Phenotyping Core and Center for Metabolism & Obesity Research at The Johns Hopkins University.

### Glucose and insulin tolerance tests

For the glucose tolerance test (GTT), mice were fasted overnight and then injected with i.p. glucose (2 g/kg body weight), whereas i.p. insulin (0.75 U/kg BW, Sigma-Aldrich) was administered to 4-h fasted mice for the insulin tolerance test (ITT). Glucose levels were then measured from the tail blood of each mouse right before the injection (0 time point) as well as at 15, 30, 60, 90, and 120 min following the i.p. injection of glucose or insulin using glucometer (Accu-chek, Roche diagnostics). The area under the curve (AUC) was generated to compare the levels of glucose among the experimental groups.

### Hyperinsulinemic–euglycemic clamp

The hyperinsulinemic–euglycemic clamp was conducted in-house as described^75^. In brief, WT and NQO1-Tg mice (after 16 weeks on diet) were fasted overnight for 15–16 h before the start of the clamp. Fasted mice were continuously infused with 0.05 μCi/min [3-^3^H]glucose over 2 h as a bolus to assess basal glucose turnover. After infusion of [3-^3^H]glucose, hyperinsulinemic–euglycemic clamping was conducted for 140 min with a primed/continuous infusion of human insulin (21 mU/kg prime over 3 min, 3 mU/kg per min infusion; Novo Nordisk) and a variable infusion of 20% dextrose to maintain euglycemia (120 mg/dL). [3-^3^H]glucose (0.1 μCi/min) was continuously infused to determine insulin-stimulated glucose uptake and endogenous glucose production after the basal period. A bolus of 2-deoxy-d-[1-^14^C]glucose (10μCi, PerkinElmer) was injected after 85 min to estimate the insulin-stimulated tissue glucose uptake. Samples were taken at 0 and 135 min for plasma fatty acid and insulin concentrations. At study completion mice were anesthetized and tissues were harvested within 3 min with liquid N2–cooled aluminum tongs and stored at –80 °C for subsequent analysis. *n* = 10 per group.

### Physical performance tests

Results are presented as time to fall from an accelerating rotarod and treadmill test according to established protocols^76^. In brief, mice were given a habituation trial on day 1 where they were placed on the rotarod at a constant speed (4 rpm) and had to remain on the rotarod for 1 min. Results shown are the average of three trials per mouse, measuring time to fall from an accelerating rotarod (4–40 rpm over 5 min). The maximum trial length was 5 min and there was a 30-min rest period between each trial. For treadmill test, results are expressed as time ran until exhaustion. Subjects were habituated at a constant speed of 4 m/min for 5 min. The following day each mouse was given a trial starting at 7 m/min for 0–3 min, 12 m/min for 3–7 min, 15 m/min for 7–25 min, and 19 m/min for 25 min. Mice (18 weeks of age) were put on SD or HFD for 14 weeks (rotarod test) and 15 weeks (treadmill test). *n* = 11–14 per group.

### Plasma biochemistry

Plasma concentrations of insulin (Crystal Chem, Elk Grove Village, IL), leptin (Thermo Fisher Scientific, Waltham, MA), adiponectin (Thermo Fisher Scientific) and FGF21 (R&D Systems, Minneapolis, MN) were measured by ELISA using microplate absorbance reader (Bio Teck Instruments), according to the manufacturer’s instructions. Plasma concentrations of ALT activity (BioVision, Inc., Milpitas, CA) and free fatty acids (Biovision) were measured by colorimetric assays, using microplate absorbance reader (Bio Teck Instruments), according to the manufacturer’s instructions.

### Quantitative RT-PCR

Total RNA was extracted using RNeasy Mini Kit (Qiagen, Germantown, MD) following the manufacturer’s instructions and quantity of RNA were analyzed using Nanodrop ND-1000 (Thermo Fisher Scientific, Waltham, MA). Isolated RNA (2000 ng) was reverse transcribed to complementary DNA using High-Capacity cDNA reverse transcription kit (Life Technologies, Carlsbad, CA). Amplification and quantification of cDNA was assessed using TaqMan^TM^ Fast Universal PCR Master Mix (Life Technologies) with TaqMan^TM^ Gene Expression Assays including mouse primers: NQO1 (Mm01253561_m1), GCLC (Mm00802655_m1), HO1 (Mm00516005_m1), SOD2 (Mm01313000_m1). Levels of transcripts were measured by quantitative real-time PCR on an ABI (Applied Biosystems, Waltham, MA) using the comparative critical threshold (∆∆Ct) method.

For quantitive PCR in epididymal white adipose tissue (eWAT) of mice, total RNA was isolated using RNeasy Lipid Tissue Mini Kit (Qiagen) and quantified using Nanodrop ND-1000. Isolated RNA (5000 ng) was reverse transcribed in a single 20 µl reaction using iScript Advanced cDNA Synthesis Kit for RT-qPCR (Bio-Rad) according to manufacturer’s instructions. The expression of target genes was assessed in triplicate reactions using SsoAdvanced Universal SYBR Green Supermix (Bio-Rad) on a QuantStudio 7 Flex Real-Time PCR System. Each 10 µl reaction mixture contained 2 µl cDNA template (diluted 1:20) and 0.5 µM of each primer (see list below). Specificity of primers was evaluated using dissociation curves and gene expression was analyzed using 2^−ΔΔCt^ method. The target genes were:

ms_Ccl-2_Forward: CCCAATGAGTAGGCTGGAGA; Reverse: TCTGGACCCATTCCTTCTTG

ms_TNF-a_Forward: ACTGAACTTCGGGGTGATCG; Reverse: GCTACAGGCTTGTCACTCGAA

ms_IL-1Ra_Forward: GTGTCCTGTTTAGCTCACCCA; Reverse: TCCCAGATTCTGAAGGCTTGC

ms_IL-10_Forward: GCGCTGTCATCGATTTCTCC; Reverse: ATGGCCTTGTAGACACCTTGG

ms_Visfatin_Forward: CTGTGGCGGGAATTGCTCTA; Reverse: GTCTTTCCCCCAAGCCGTTA

Housekeeping gene: ms_Ppia_Forward: GACCAAACACAAACGGTTCC; Reverse: CATGCCTTCTTTCACCT.

### Western blot analysis

Tissues were homogenized using metal beads in a Tissue Lyser (Qiagen). Samples were incubated on ice for 30 min and then centrifuged at 4 °C for 30 min at 14,000 rpm. The supernatant was transferred to a new tube, protein concentration was determined by the BCA protein assay kit (Thermo Fisher Scientific). Equal amounts of lysates were resolved on SDS-PAGE gels and electrophoretically transferred to nitrocellulose membranes (Bio-Rad, Hercules, CA). Membranes were blocked with 5% (w/v) nonfat milk, and then incubated with a specific primary antibody to each target protein overnight at 4 °C. Primary antibodies were directed against a number of proteins as denoted in Supplementary Table 8. Following separate steps of washing to remove unbound antibodies, membranes were probed with a species-specific HRP-conjugated secondary antibody for 1 h. Image was detected by Immobilon Western Chemiluminescent HRP Substrate Kit (Millipore, Burlington, MA). Equivalent loading was further assessed by ponceau S staining. All blots or gels depicted in a given figure panel derive from the same experiment in which samples were processed in parallel.

### NQO1 activity

NQO1 enzymatic activity was determined using 2,6-dicholorphenolindophenol (DCPIP) (Sigma-Aldrich) as the two-electron acceptor, as described with modifications^77^. In brief, the reaction mix was composed of 25 mM Tris–HCl, pH 7.4, 0.01% Tween-20, 0.2 mM NADH, and 80 µM DCPIP in the presence or absence of 40 µM dicumarol. The reduction of DCPIP was monitored at 600 nm at 25 °C for 3 min. The dicumarol-inhibitable NQO1 activity was calculated using an extinction coefficient of 21.0 mM^–1^ cm^–1^.

### SIRT3 activity

SIRT3 enzymatic activity was assayed in liver extracts following the manufacturer’s instructions (Enzo Life Sciences, Farmingdale, NY) with slight modifications^28^.

### GOT2 and CRAT activities

Both activities were measured in liver extracts using mouse Aspartate aminotransferase mitochondrial ELISA kit (#MBS761622, MyBioSource, San Diego, CA) and mouse Carnitine *O*-acetyltransferase ELISA kit (#MBS282006, MyBioSource), according to manufacturer’s instructions.

### Glyceride quantification

Liver tissue was homogenized in 1 mL methanol and the lipids were extracted with 2 mL of chloroform. After vortexing, samples were centrifuged at 1000 x *g*. The organic phase was isolated, and water was added to further separate pooled organic phases. After centrifugation (1000 x *g*, 5 min) the lower phase was dried. SPE columns (Waters, Milford, MA) were used to separate lipid species, according to a published lipid separation method^78^, and free glycerol content was determined by colorimetric assay (Sigma-Aldrich).

### Tissue histology

To examine hepatic pathological changes, small liver specimens were collected from Keap1 *fl/fl* (control, *n* = 7; WD, *n* = 8) and KO (control, *n* = 8; WD, *n* = 7) mice following feeding for 26 weeks. Liver specimens was immediately fixed in 10% neutral formalin and subjected to hematoxylin and eosin (H&E) staining by Reference Histology Core in Johns Hopkins University (Baltimore, MD). For the NQO1-Tg project, small specimens of liver and fat tissue, fixed in 4% paraformaldehyde, were embedded into paraffin blocks for sectioning and then mounting on glass covers (Histoserv, Inc., Germantown, MD). H & E staining was performed to evaluate tissue architecture while Periodic Acid Schiff (PAS) reagent was used to stain for glycogen according to well-established protocols. Scoring of liver damage and glycogen content (PAS staining) was performed on at least 3 fields/animal (*n* = 6/group).

### Sucrose fractionation

Quadriceps (2.4 mg) pooled from five animals of each group were homogenized with a Dounce homogenizer in 1 mL polysome extraction buffer (PEB): 20 mM Tris–HCl pH 7.5, 100 mM KCl, 5 mM MgCl_2_, 0.3 % NP-40, 60 U/mL RiboLock RNase Inhibitor (Thermo Fisher Scientific), 100 μg/mL cycloheximide, 5 µM FAD, cOmplete EDTA-free protease inhibitor cocktail and phosphatase inhibitor cocktail (Sigma-Aldrich). Samples were incubated on ice for 15 min and then centrifuged at 12,000 r.p.m. for 10 min at 4 °C. Cytoplasmic extracts with equal amounts of RNA were loaded on a 15–50% sucrose gradient and centrifuged at 4 °C in a SW 41 Ti Beckman rotor for 3 h 30 min at 39,000 r.p.m. as described^43^. Twelve fractions were collected using a fraction collector (Brandel) and monitored by optical density measurement (A254). Equal amount of each fraction was mixed with 4x Laemmli Sample Buffer (Bio-Rad) and resolved on SDS-PAGE followed by immunoblot analysis.

### Immunoprecipitation

Skeletal muscle homogenates were prepared in PEB as described in the sucrose fractionation procedure. The total protein concentration in the homogenate was determined by BCA assay (Pierce) and adjusted to 3 mg/mL with homogenization buffer. SureBeads Protein G Magnetic Beads (Bio-Rad) were conjugated with 8 μg of goat anti-NQO1 antibody (Abcam) or IgG goat control (Vector labs) following manufacturer’s instruction. Homogenates were first incubated with 30 µl goat IgG-conjugated beads for 30 min at 4 °C under gentle agitation. Beads were discarded and pre-cleared homogenates containing 1 mg of protein were incubated for 3 h at 4 °C with 100 µl of magnetic bead-conjugated antibodies under rotation. Where indicated, incubation was performed with the addition of 10 µl DMSO, 100 µM beta-lapachone, 100 µM dicoumarol, or 1 µg RNase A. Beads were magnetized and washed five times with 50 mM Tris, pH 7.4, 150 mM NaCl, 1 mM MgCl_2_, 0.05% Nonidet P-40, 5 µM FAD. Washing steps were performed by incubating beads with 1 mL washing buffer for 5 min at 4 °C. Samples were eluted incubating beads in 2x Laemmli Sample Buffer without reducing agent, at 70 °C for 10 min on a shaking platform set at 1400 r.p.m.

### Acetylomics

#### Liquid chromatography–mass spectrometry (LC-MS/MS) analysis of acetylated proteins, including accurate mass and retention time (AMRT) library generation

Enriched acetyl-Lys peptides were pooled by sample group and were analyzed as described elsewhere^79^. Briefly, samples were loaded onto a 2 cm PepMap 100 nanoviper trapping column and resolved via 2.0 µm Acclaim PepMap RSLC column (Thermo Scientific) using a 1290 Infinity II LC with nanoadapter (Agilent). Chromatographic buffers consisted of water + 0.1% formic acid (A) and 90% acetonitrile + 0.1% formic acid (B). Samples were resolved using 330 nL/min using a gradient of 3–8% B for 2 min, 8–26% B for 26 min, and 26–40% B for 2 min for a 30 min gradient at 60 °C and was followed by a column wash at 75% B over 5 min. A 6550 Q-TOF and nano source (Agilent) was utilized with intensity-dependent CID MS/MS for peptide analysis and SpectrumMill (Agilent) was employed for data analysis as detailed previously^79^. Peptide searches allowed for up to 4 missed tryptic cleavages and included fixed carbamidomethyl (C) and variable deamidated (NQ), oxidation (M), and acetyl (K) modification. The AMRT library was generated using a minimum peptide score of 8 and a peak intensity of 50%. MS-only quantitation was performed in positive ion polarity (260–1700 *m*/*z*) at a 1.5 spectra/second scan rate in MS-only mode (Agilent 6550 Q-TOF). Acetyl-Lys peptides were analyzed as described above.

MS quantitation analysis was performed using Profinder V.B.10.00 software (Agilent) and the AMRT library was used for batch feature extraction in a targeted manner as previously described^79^. Quantitative MS-only data files were warped or retention times pre-aligned to the WT-HFD pooled MS/MS data file as a reference using the polynomial interpolation method in Profinder. Acetyl-Lys extraction and alignment was analyzed in Mass Profiler Professional V.14.8 (Agilent) for quantitation. Each sample was normalized by external scalar to the Procal retention time standards summed extracted areas found across all samples to control for technical variance due to the mass spectrometer. The full proteomics dataset has been deposited to the ProteomeXchange Consortium via the PRIDE [1] partner repository with dataset identifier PXD015070.

Statistical analysis was performed as described previously^79^. Features were limited to those only found in 100% of 1 of 2 conditions for comparison. Acetylated peptides were then applied to volcano plots via moderated *t*-test and significance (fold-change ≤ or ≥ 1.5 and *p*-value < 0.05). Benjamini–Hochberg analysis with and without multiple-testing correction was applied to acquire a list of acetyl-peptides that were significantly different and allowed for the examination of how acetylation modifications impact individual proteins. Two-way ANOVA was used to examine genetic differences vs. dietary differences for individual lysine acetylation sites on a given protein.

### Metabolomics

Untargeted metabolomics analysis on liver, skeletal muscle, serum and visceral fat extracts, and serum obtained at the time of sacrifice was performed at Metabolon, Inc. (Research Park Triangle, NC, USA). Subsequently, metabolite profiles were analyzed using MetaboAnalyst version 4.0^36,37^, utilizing univariate and multivariate built-in analytical methods from modules of this web-based platform, as specified.

### Quantification and statistical analysis

No statistical methods were used to predetermine sample size. Investigators were not blinded to allocation during experiments and outcome assessment. Results are expressed as mean ± SEM or graphically represented as boxplots. Statistical significance was determined by unpaired two-tailed Student’s *t*-test using statistical software (Prism 8.0, GraphPad). Differences were considered statistically significant when *p*-value < 0.05.

### Reporting summary

Further information on research design is available in the Nature Research Reporting Summary linked to this article.

## Supplementary information

Supplemental Material

Full blots

Reporting summary

Table S1_metabolomics

Table S2_lipid species

Table S3_acetylomics

## Data Availability

The data sets generated during and/or analyzed during the current study are available from the corresponding author, Rafael de Cabo (decabora@mail.nih.gov), on reasonable request. The metabolomics data have been deposited in the MetaboLights database^80^ with the following accession number MTBLS2085 (https://www.ebi.ac.uk/metabolights/MTBLS2085). RAW MS data from our skeletal muscle proteome analysis have been deposited to the ProteomeXchange Consortium (http://proteomecentral.proteomexchange.org) via the PRIDE partner repository with the dataset identifier PXD015070.
