# Supplementary material for: NQO1 protects obese mice through improvements in glucose and lipid metabolism
Source: NPJ Aging Mech Dis. 2020 Nov 19;6:13. doi: 10.1038/s41514-020-00051-6 (PMC7678866; doi:10.1038/s41514-020-00051-6)
Supplement: Supplementary file 1 — Supplemental Material [file 41514_2020_51_MOESM1_ESM.pdf]

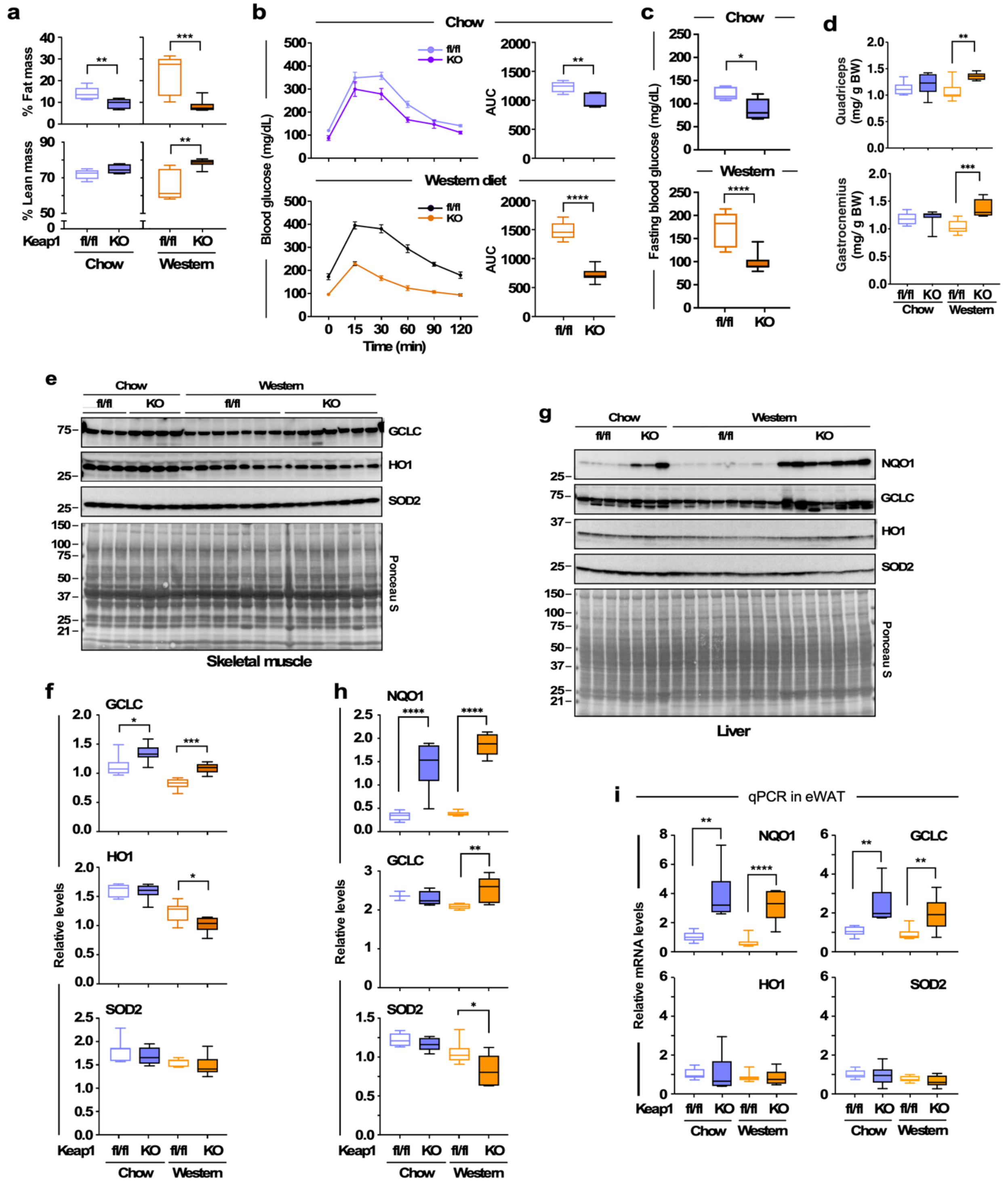

Supplementary Fig. 1. Physiology of Nrf2 knockdown in mice. a Measures of percent body fat and lean mass by NMR. n = 6-8 per group. **b** Left panels, blood glucose levels (mg/dL) during an oral glucose tolerance test; right panels, area under the curve (AUC). n = 5-10 per group. **c** Fasting blood glucose

levels at baseline (see panel B). **d** Western blot for GCLC, HO1, and SOD2 in skeletal muscle extracts. Molecular mass markers (kDa) are depicted on the left. **e** Densitometric quantification after normalization with Ponceau S staining of the membrane. n = 7-8 per group. **f** Western blot for NQO1, GCLC, HO1, and SOD2 in liver extracts. Molecular mass markers (kDa) are depicted on the left. **g** Densitometric quantification after normalization with Ponceau S staining of the membrane. n = 7-8 per group. **h** Quantitative PCR analysis for NQO1, GCLC, HO1, and SOD2 mRNA levels in epididymal WAT extracts. All data are expressed as means ± SEM. Comparison by two-tailed t-test was performed unless otherwise specified. *, **, ***, ****p < 0.05, 0.01, 0.001, and 0.0001 versus fl/fl littermate controls. Related to Fig. 1.

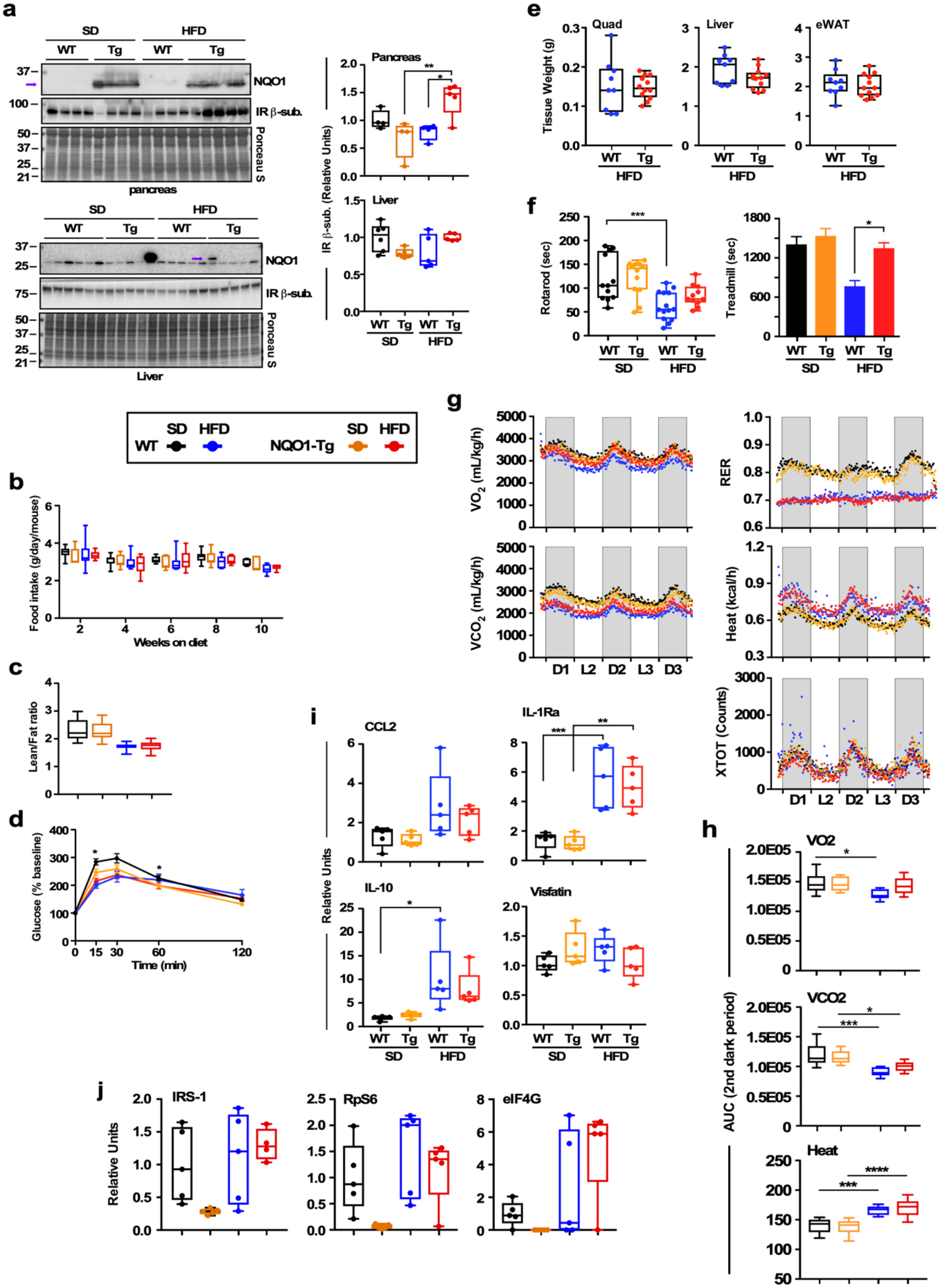
a
SD
HFD
WT
Tg
NQO1
IR β-sub.
Ponceau S
pancreas
Liver
Pancreas
IR β-sub. (Relative Units)
b
Food intake (g/day/mouse)
Weeks on diet
c
Lean/Fat ratio
d
Glucose (% baseline)
Time (min)
e
Quad
eWAT
Tissue Weight (g)
f
Rotarod (sec)
Treadmill (sec)
g
VO2 (mL/kg/h)
VCO2 (mL/kg/h)
RER
Heat (kcal/h)
XTOT (Counts)
D1
L2
D2
L3
D3
NQO1-Tg
h
VO2
VCO2
Heat
AUC (2nd dark period)
i
CCL2
IL-1Ra
IL-10
Visfatin
Relative Units
j
IRS-1
RpS6
eIF4G

Supplementary Fig. 2. Physiology of NQO1 transgenesis. a *Left panels*, immunoblots for NQO1 and IR β-subunit from pancreas and liver homogenates. Purple arrows denote the rat NQO1 transgene. Ponceau S staining of the membrane is shown and the molecular weight protein standards (kDa) are depicted on the left. *Right panels*, densitometric quantification of the IR β-subunit. Values are represented as box plots, n=5-6 per group. **b** Food intake for the first ten weeks on diet. Values are represented as box plots, n=22-36 per group. **c** Lean-to-fat ratio after 14 weeks on diet. Values are represented as box plots, n=12-16 per group. **d** Oral glucose tolerance test (OGTT) was performed on 11-mo-old mice (5 weeks on diet). Values represent means ± SEM, n=10-12 mice per group. **e** Organ weight at sacrifice. **f** Physical performance assessed by rotarod and treadmill endurance tests after 14-15 weeks on diet. **g** Eight-month-old mice (8-10 weeks on diet) were placed into metabolic cages to measure the respiratory exchange ratio (RER) as detailed in the method online, n=6-13 mice per group. **h** Area under the curve (AUC) for VO_2, VCO_2, and heat production. **i** qPCR analysis in eWAT. Related to Figure 3d. **j** Densitometric quantitation of IRS-1, RpS6 and eIF4G proteins from skeletal muscle homogenates derived from WT and NQO1-Tg mice fed SD or HFD. Immunoblots are shown in Figures 3a and 4b. Values are represented as box plots with individual values, n=5 per group.

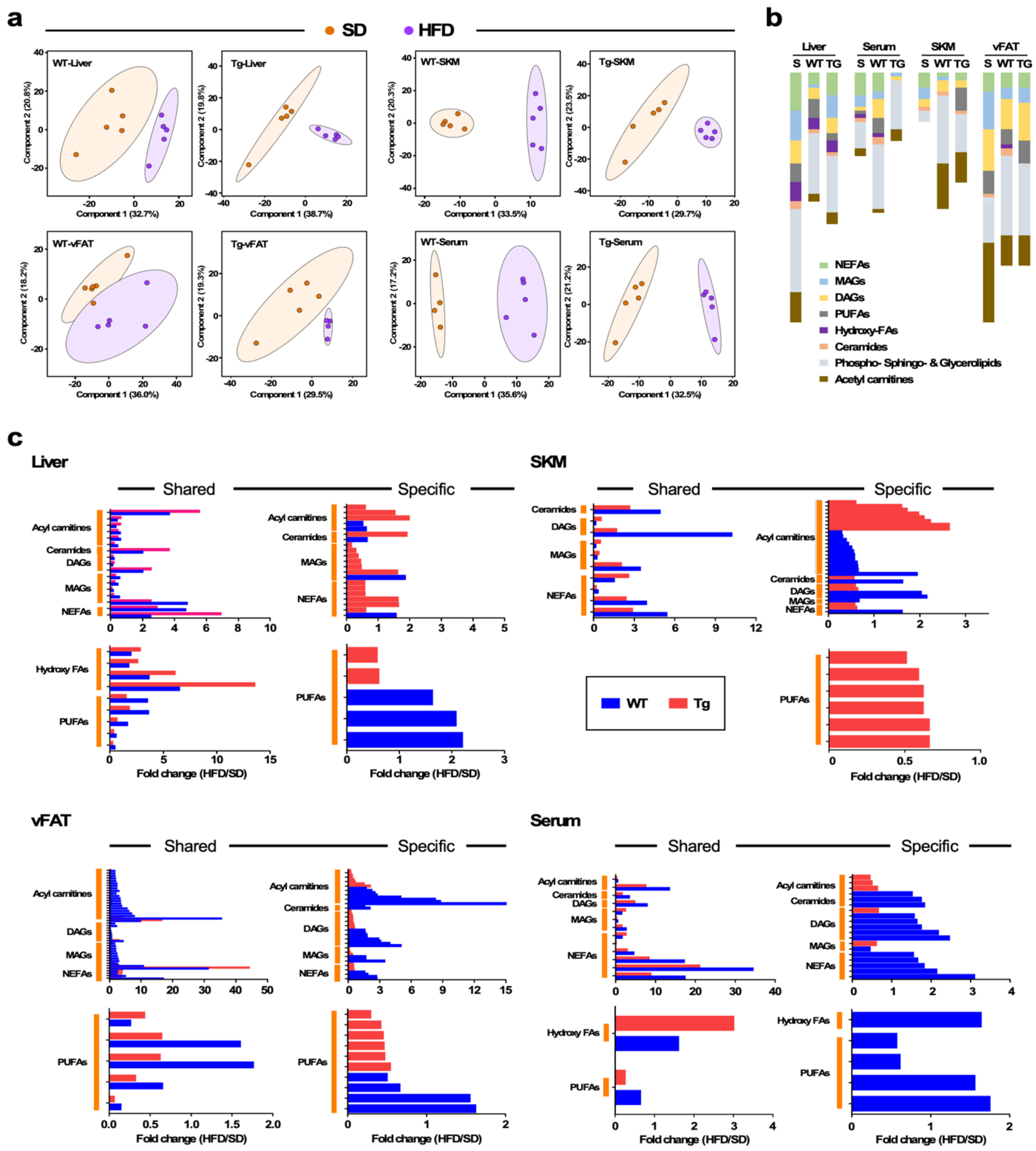

Supplementary Fig. 3. Metabolomic analysis in various organs and serum. a Liver, skeletal muscle (SKM), visceral adipose tissue (vFAT) and serum metabolite profiles from WT and NQO1-Tg mice fed SD (orange symbols) or HFD (purple symbols) were analyzed by Partial Least Square Discriminant Analysis (PLSDA). A statistically significant degree of separation is observed between diet groups. The ellipses correspond to 95% confidence intervals for a normal distribution. Each principal component is labeled with the corresponding percent values. **b** View of the distribution of the eight lipid species

significantly enriched in the indicated tissues and serum. S, lipids that were shared between WT and Tg mice. **c** Abundance of various lipid species that were either shared between WT (blue bars) and NQO1-Tg (red bars) mice or unique to each genotype. Results are expressed as fold change in metabolite levels in HFD vs. SD. Related to Fig. 4a and 4b**.**

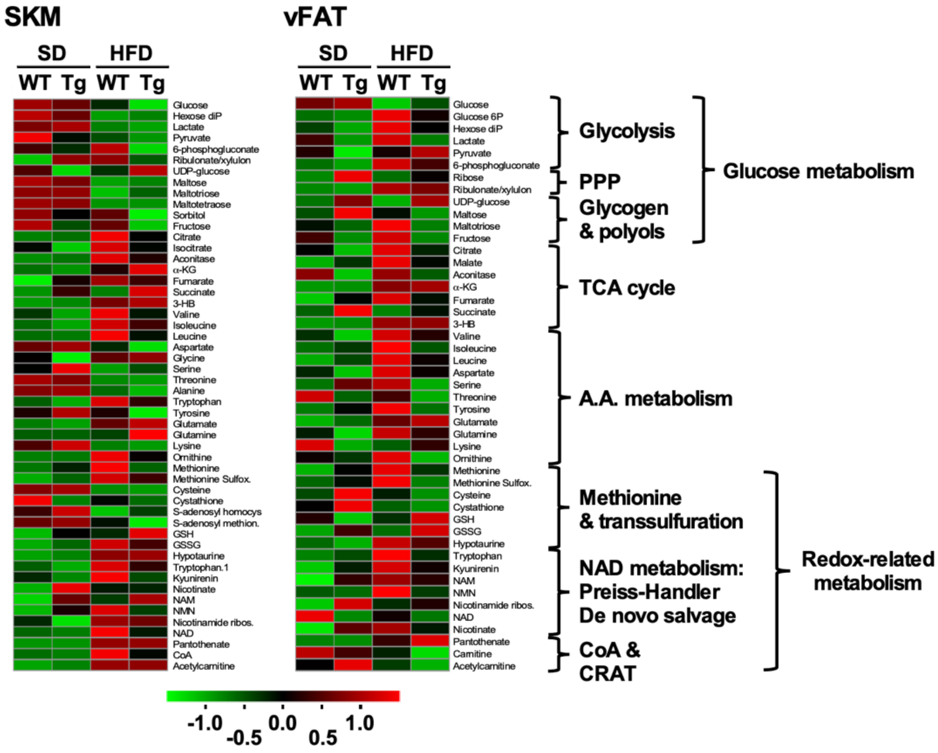

Supplementary Fig. 4. Heatmap visualization of metabolites in skeletal muscle (SKM, left) and visceral adipose tissue (vFAT, right) of WT and NQO1-Tg mice fed SD or HFD. Upregulation (red font), down regulation (green font). Related to Fig. 4a.

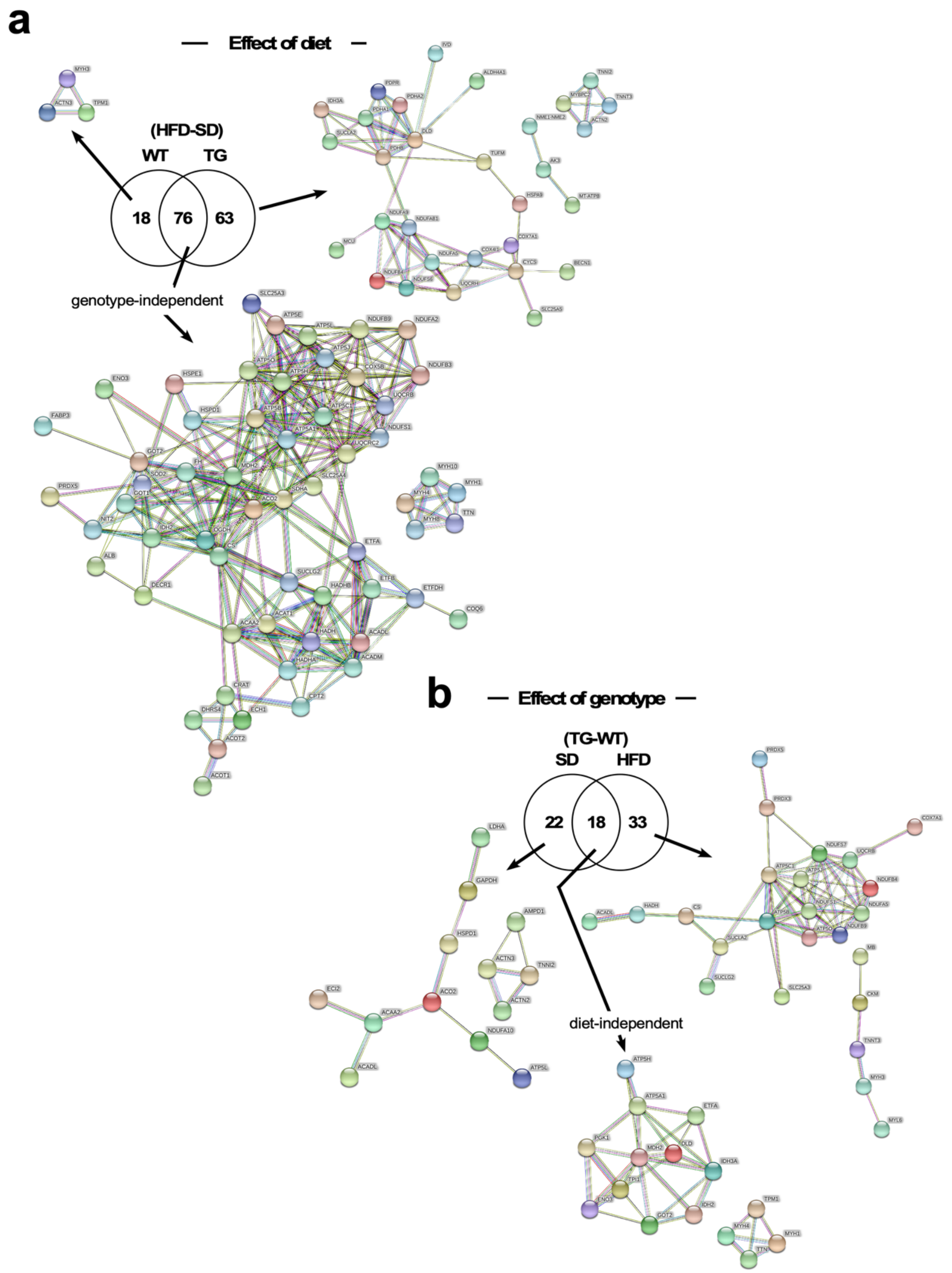

Supplementary Fig. 5. Effects of diet (a) and genotype (b) on the interaction between lysine acetylated proteins in skeletal muscle of WT and NQO1-Tg mice. The data derived from the indicated pairwise comparisons was analyzed with STRING functional network association (http://string-db.org). Related to Fig. 5 and Supplementary Table 4.

Supplementary Table 1. List of shared and genotype-specific metabolites differentially influenced by HFD vs. SD in liver, serum, skeletal muscle, and visceral adipose tissue of WT littermates and NQO1-Tg (TG) mice (see Excel sheet). Related to Fig. 4.

Supplementary Table 2. List of various lipid species differentially impacted by HFD vs. SD in liver, serum, skeletal muscle, and visceral adipose tissue of WT littermates and NQO1-Tg (TG) mice (see Excel sheet). For each tissue, lipids that are common between the two genotypes and unique for each genotype are depicted. Related to Fig. 4 and Supplementary Fig. 3c.

Supplementary Table 3a. List of lysine-acetylated proteins impacted by the rat NQO1 transgene in skeletal muscle of mice fed SD and HFD. (See Excel sheet, tab 1). Related to Fig. 5b.

Supplementary Table 3b. List of lysine-acetylated proteins impacted by the diet (HFD vs. SD) in skeletal muscle of WT and NQO1-Tg mice. (See Excel sheet, tab 2). Related to Fig. 5b.

Supplementary Table 4. Protein-protein interaction network statistics derived from String (http://string-db.org). Related to Fig. 5b and Supplementary Fig. 5.

	(HFD-SD)			(Tg-WT)		
	WT unique	Tg unique	shared	SD unique	HFD unique	shared
Number of nodes	8	40	65	21	28	18
Number of edges	3 (exp. 0)	58 (exp. 4)	271 exp. 12)	13 (exp. 1)	55 (exp. 3)	33 (exp. 1)
Avg. node degree	0.75	2.9	8.34	1.24	3.93	3.67
Avg. local clustering coeff.	0.375	0.564	0.586	0.349	0.522	0.662
PPI enrichment p-value	0.0102	<1.0e-16	<1.0e-16	9.86e-12	<1.0e-16	<1.0e-16

HFD, high-fat diet; SD, standard diet; WT, wild-type littermates; Tg, NQO1 transgenic mice.

Supplementary Table 5. Top biological processes (GO terms) impacted in the indicated pairwise comparisons. Related to Fig. 5b and Supplementary Fig. 5.

GO term	Description	FDR (-log10)
**(HFD-SD) unique for WT**		
0046034	ATP metabolic process	3.54
0030049	Muscle filament sliding	3.54
0009167	Purine ribonucleoside monophosphate metabolic process	3.54
0006936	Muscle contraction	3.54
0006941	Striated muscle contraction	3.39
**(HFD-SD) unique for Tg**		
0006091	Generation of precursor metabolites and energy	18.15
0045333	Cellular respiration	18.00
0009150	Purine ribonucleotide metabolic process	17.91
0017144	Drug metabolic process	16.80
0055114	Oxidation-reduction process	16.32
**(HFD-SD) shared in both genotypes**		
0044281	Small molecule metabolic process	39.64
0006091	Generation of precursor metabolites and energy	29.60
0055114	Oxidation-reduction process	25.65
0017144	Drug metabolic process	22.73
0009117	Nucleotide metabolic process	22.34
**(Tg-WT) unique for SD**		
0055114	Oxidation-reduction process	3.28
0044281	Small molecule metabolic process	3.28
0032787	Monocarboxylic acid metabolic process	3.28
0030049	Muscle filament sliding	3.28
0019752	Carboxylic acid metabolic process	3.28
**(Tg-WT) unique for HFD**		
0017144	Drug metabolic process	17.01
0006091	Generation of precursor metabolites and energy	15.39
0044281	Small molecule metabolic process	15.14
0046034	ATP metabolic process	14.31
0055114	Oxidation-reduction process	13.63
**(Tg-WT) shared by both diets**		
0006091	Generation of precursor metabolites and energy	9.53
0017144	Drug metabolic process	9.46
0044281	Small molecule metabolic process	8.84
0009117	Nucleotide metabolic process	8.47
0046034	ATP metabolic process	7.55

Supplementary Table 6. List of Acetylated Peptides Differentially Influenced by Diet x Genotype Interaction using a Two-way ANOVA. Related to Fig. 5.

Description; UniProt ID; Peptide sequence; Lysine modified;	Diet x genotype	Diet	Genotype
Troponin T, fast skeletal muscle; Q9QZ47; QkYDITTLR; K239k; 99	0.00244	0.208	0.0196
Succinyl-CoA ligase [GDP forming] subunit α, mitochondrial; Q9WUM5; GGQkHLGLPVFNTVK; K94k; 15.617	0.0368	0.000279	0.478
Succinyl-CoA ligase [GDP forming] subunit α, mitochondrial; Q9WUM5; IGIMPGHIHkK; K192k; 0	0.0443	0.000346	0.408
Succinyl-CoA ligase [ADP forming] subunit β, mitochondrial; Q9Z219; AVSSQMIGQkLITK; K139k; 4.252	0.0133	0.00881	0.0590
Succinyl-CoA ligase [ADP forming] subunit β, mitochondrial; Q9Z219; SSDEAYAIAkK; K88k; 2.461	0.0272	0.00105	0.316
Stress-70 protein, mitochondrial; P38647; NVPFkIVR; K143k; 99	0.0215	0.000390	0.157
Pyruvate dehydrogenase E1 component subunit β, mitochondrial; Q9D051; DFLIPIGkAK; K227k; 0.768	0.0285	0.000294	0.0384
# Pyruvate dehydrogenase E1 component subunit α, mitochondrial; P35486; SKSDPIMLLkDR; K321k; 14.682	0.0124	0.00581	0.0971
Polycystic kidney disease protein 1-like 3; Q2EG98; FFLHRAIWk; K1702k; 99	0.0124	0.617	0.0421
NAD(P)H dehydrogenase [quinone] 1; Q64669; NkFGLSVGHHLGK; K251k; 15.61	0.000409	0.000157	1.59E-17
Myosin-3; P13541; IAEKDEEIEQLkR; K1588k; 11.828	0.0251	0.00513	0.237
Myosin-1; Q5SX40-Q5SX39-P13542; VKELTYQTEEDRkNVLR; K1866k; 14.589	0.0353	0.158	0.0209
# Myosin-1; Q5SX40-Q5SX39; LYEQHLGkSNNFQKPKPAK; K562k; 4.91	0.00505	0.000538	0.0313
Myosin-1; Q5SX40-P13542; VRELEGEVENEQkR; K1838k; 99	0.0106	0.000377	0.374
Myosin-1; Q5SX40; IHELEkIK; K1535k; 3.535	0.0288	0.000188	0.549
Myoglobin; P04247; THPETLDkFDK; K43k; 6.771	0.00568	0.431	0.0713
Malate dehydrogenase, mitochondrial; P08249; KGEDFVkNMK; K335k; 4.538	0.0377	0.000633	0.552
Malate dehydrogenase, mitochondrial; P08249; KGLEkNLGIGK; K301k; 6.471	0.0453	0.0000674	0.226
# Isovaleryl-CoA dehydrogenase, mitochondrial; Q9JHI5; HTISkFLQENLAPK; K56k; 14.357	0.0226	0.0141	0.000502
GTP:AMP phosphotransferase, mitochondrial; Q9WTP7; ITkHFELK; K29k; 9.174	0.00585	0.0000536	0.228
Eukaryotic translation initiation factor 5A-1; P63242-Q8BGY2; IVEMSTSkTGK; K47k; 3.97	0.0477	0.045	0.290
Electron transfer flavoprotein subunit β; Q9DCW4; VKPDkSGVVTDGVK; K26k; 3	0.0356	0.0000661	0.209
# Electron transfer flavoprotein subunit α, mitochondrial; Q99LC5; VVQDLCkVAGVAK; K69k; 11.202	0.0469	0.0000123	0.0382
# Dehydrogenase/reductase SDR family member 4; Q99LB2; EkLITTALK; K99k; 13.601	0.0408	0.0000014	0.0379
Cytochrome c, testis-specific; P00015-P62897; MIFAAGIkK; K87k; 3.079	0.0395	0.000271	0.626
Cytochrome c oxidase subunit 5B, mitochondrial; P19536; EDPNLVPSISNkR; K85k; 99	0.0414	0.000101	0.285
Cytochrome b-c1 complex subunit 7; Q9D855; SAVSASSkWLDGFRK; K12k; 3.061	0.0448	0.00966	0.000823

Cytochrome b-c1 complex subunit 6, mitochondrial; P99028; DHCVAHkLFK; K83k; 7.912	0.0395	0.00168	0.739
Creatine kinase M-type; P07310; HNNHMAkVLTPDLYNK; K32k; 10.381	0.00615	0.00189	0.814
Citrate synthase, mitochondrial; Q9CZU6; IVPNILLEQGkAK; K393k; 3.042	0.0351	0.110	0.211
Beta-enolase; P21550; IGAEVYHHLk; K193k; 99	0.0299	0.0341	0.510
Alpha-actinin-2; Q9JI91; TPEkTMQAMQK; K311k; 5.808	0.00315	0.461	0.218
Acyl-CoA thioesterase 13; Q9CQR4; LICEMkVEEQHTNK; K43k; 1.726	0.0126	0.0000155	0.0978
ATP synthase subunit gamma, mitochondrial; Q91VR2; GLCGAIHSSVAkQmK; K112k M114m; 1.331	0.0397	0.0000155	0.854
ATP synthase subunit gamma, mitochondrial; Q91VR2; GLCGAIHSSVAkQMK; K112k; 1.522	0.0132	0.000486	0.250
ATP synthase subunit beta, mitochondrial; P56480; VVDLLAPYAkGGK; K198k; 0.5	0.0341	0.0454	0.237
ATP synthase subunit beta, mitochondrial; P56480; IMNVIGEPIDERGPIkTK; K159k; 0.799	0.0348	0.0103	0.0150
ATP synthase subunit alpha, mitochondrial; Q03265; GYLDkLEPSK; K498k; 10.192	0.0153	0.0350	0.0147
ATP synthase subunit alpha, mitochondrial; Q03265; GYLDKLEPSKITk; K506k; 0	0.0317	0.00307	0.0313
ATP synthase subunit alpha, mitochondrial; Q03265; AMkQVAGTMK; K427k; 16.046	0.0104	0.898	0.0916
ATP synthase protein 8; P03930; VKTPWELkWTK; K54k; 4.464	0.0332	0.00178	0.0639
40S ribosomal protein S3a; P97351; EVVNkLIPDSIGk; K187k K195k; 99	0.0332	0.147	0.793
2,4-deinoyl-CoA reductase, mitochondrial; Q9CQ62; FEkEMIDRIPCGR; K260k; 99	0.0138	0.0000025	0.119
10 kDa heat shock protein, mitochondrial; Q64433; kFLPLFDR; K8k; 99	0.0266	0.0000099	0.109

#, Selected lysine-modified proteins graphically represented as box plots in Fig. 5d.

Supplementary Table 7. Composition of standard chow control diet and Western diet.

	**Chow control diet (3.0 kcal/g)**	**Western diet* (4.67 kcal/g)**
	% kcal from	% kcal from
Protein	32	17
Carbohydrate	54	43
Fat	14	40
Ingredient		*G*
Casein		195
DL-Methionine		3
Corn Starch		50
Sucrose		350
Lodex10		100
Solka Floc, FCC200		50
Corn Oil		10
Butter, Anhydrous		200
S10001A		17.5
Calcium Phosphate, Dibasic		17.5
Calcium Carbonate, Light, USP		4
Choline Bitartrate		2
V10001C		1
Ethoxyquin		0.04
Cholesterol, NF		1.5
Total		1001.54

*Western diet: D12079B, Research Diets

Supplementary Table 8. List of primary antibodies used for Western blot analyses.

Antigen	Residue modified	Supplier	Cat. number	Identifier
NQO1	Total	Abcam	#ab2346	RRID: AB_302995
mTOR	Total	Cell Signaling	#2972	RRID: AB_ 330978
mTOR	Serine 2448	Cell Signaling	#2971	RRID: AB_ 330970
S6K1	Total	Cell Signaling	#9202	RRID: AB_ 331676
S6K1	Threonine 389	Cell Signaling	#9234	RRID: AB_ 2269803
RpS6	Total	Cell Signaling	#2217	RRID: AB_ 331355
RpS6	Serine 240/244	Cell Signaling	#2215	RRID: AB_331682
4E-BP1	Total	Cell Signaling	#9644	RRID: AB_2097841
4E-BP1	Threonine 37/46	Cell Signaling	#2855	RRID: AB_560835
IR β-sub.	Total	Cell Signaling	#3025	RRID: AB_ 2280448
IRS-1	Total	Cell Signaling	#3407	RRID: AB_ 2127860
IRS-1	Serine 1101	Cell Signaling	#2385	RRID: AB_ 330363
AS-160	Serine 588	Cell Signaling	#8730	RRID: AB_10860251
eIF4G	Total	Cell Signaling	#8701	RRID: AB_11178378
GCLC	Total	Abcam	#ab41463	RRID: AB_941717
HO1	Total	Abcam	#ab13243	RRID: AB_299790
SOD2	Total	Abcam	#ab13533	RRID: AB_300434
Acetyl-Lysine	Lysine	Cell Signaling	#13416	RRID:
ACC	Total	Cell Signaling	#3676	RRID:AB_2219397
FASN	Total	Cell Signaling	#3180	RRID:AB_22100796
ACLY	Total	Cell Signaling	#4332	RRID:AB_2223744
Lipin 1	Total	Cell Signaling	#14906	RRID:AB_2708644
