# Supplementary material for: NQO1 protects obese mice through improvements in glucose and lipid metabolism
Source: NPJ Aging Mech Dis. 2020 Nov 19;6:13. doi: 10.1038/s41514-020-00051-6 (PMC7678866; doi:10.1038/s41514-020-00051-6)

Full-length blots

Figure 3a

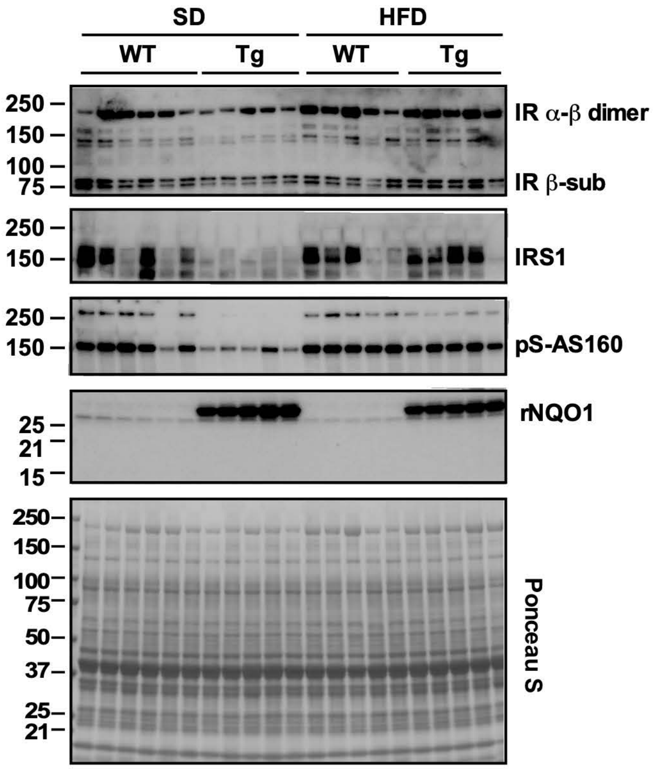

Figure 4b

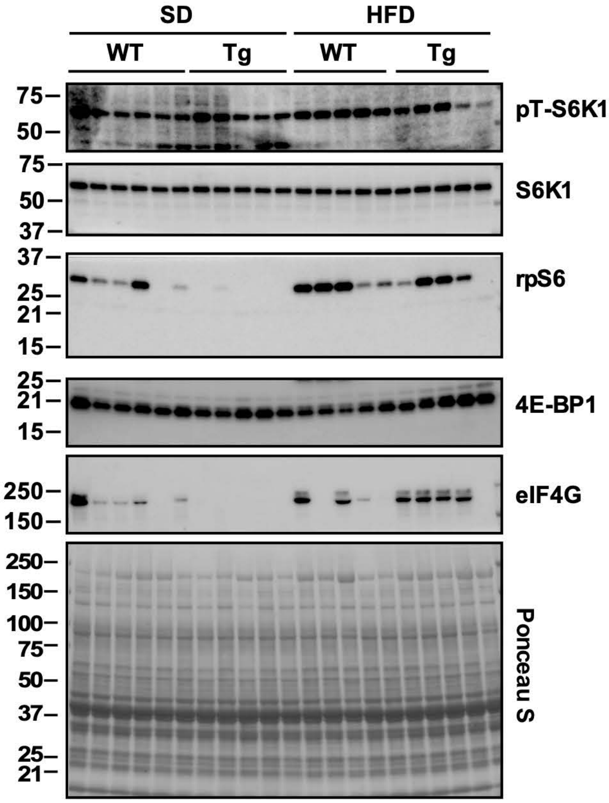

Figure 3e

eWAT lipogenic enzymes

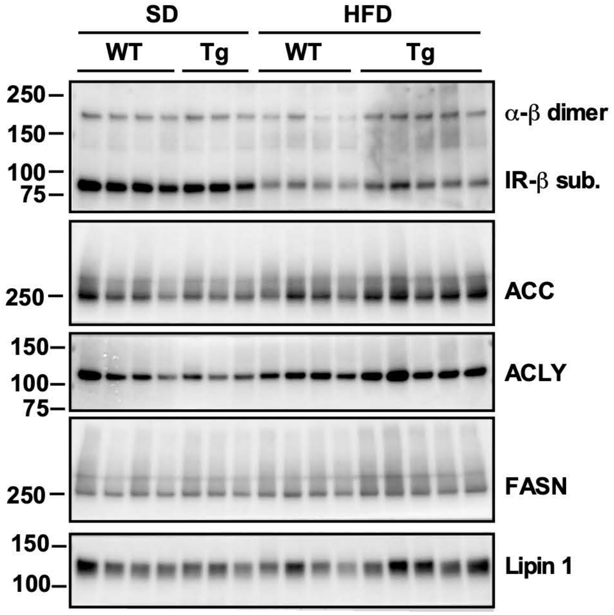

Figure 4c

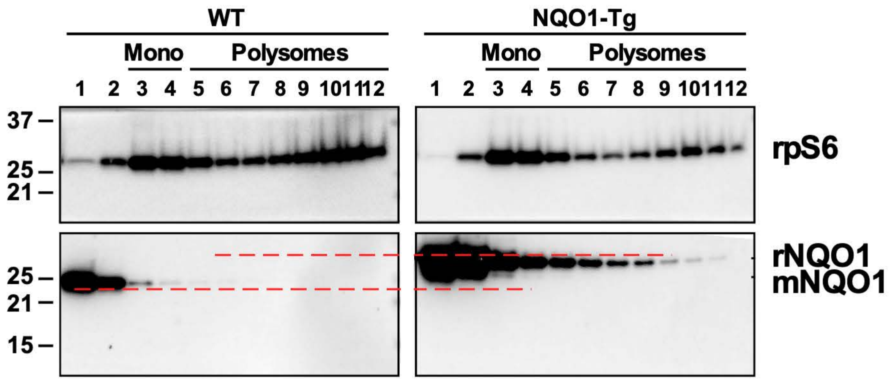

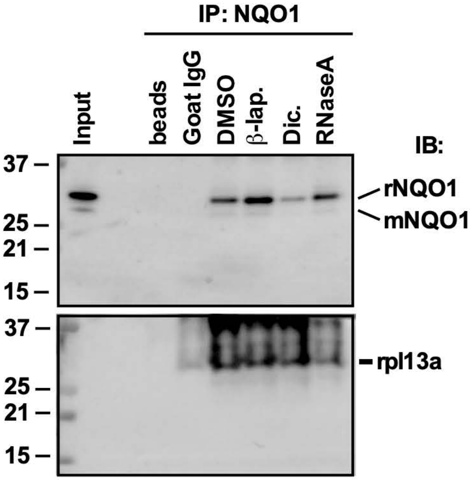

Figure 4e

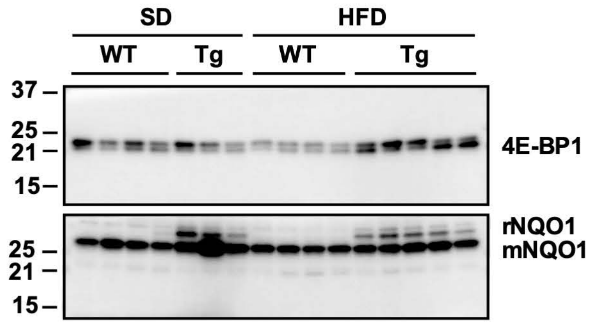

Figure 4d

Figures S1e and S1g: The Ponceau S-stained nitrocellulose membranes were destained and cut into small strips for the concurrent detection of proteins of various MWs.

Figure S2a

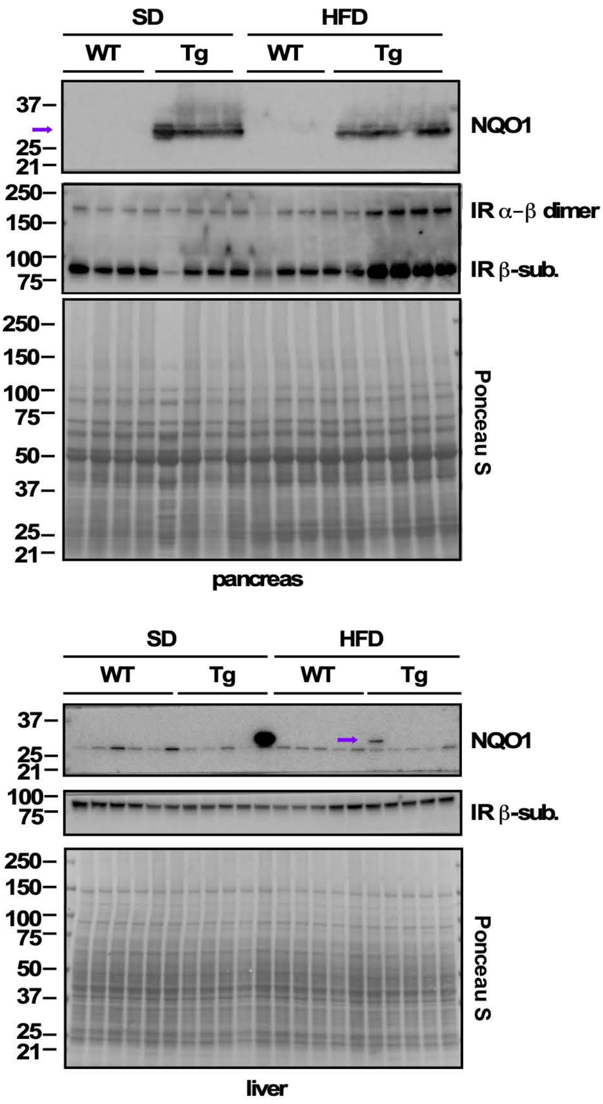

Supplement: Supplementary file 2 — Full blots [file 41514_2020_51_MOESM2_ESM.pdf]
