# Supplementary material for: NQO1 protects obese mice through improvements in glucose and lipid metabolism
Source: NPJ Aging Mech Dis. 2020 Nov 19;6:13. doi: 10.1038/s41514-020-00051-6 (PMC7678866; doi:10.1038/s41514-020-00051-6)
Supplement: Supplementary file 3 — Reporting summary [file 41514_2020_51_MOESM3_ESM.pdf]

nature research

Corresponding author(s): NPJAMD-00262R1

Last updated by author(s): Sep 16, 2020

Reporting Summary

Nature Research wishes to improve the reproducibility of the work that we publish. This form provides structure for consistency and transparency in reporting. For further information on Nature Research policies, see Authors & Referees and the Editorial Policy Checklist .

Statistics

For all statistical analyses, confirm that the following items are present in the figure legend, table legend, main text, or Methods section.

n/a	Confirmed	
☐	☒	The exact sample size (*n*) for each experimental group/condition, given as a discrete number and unit of measurement
☐	☒	A statement on whether measurements were taken from distinct samples or whether the same sample was measured repeatedly
☐	☒	The statistical test(s) used AND whether they are one- or two-sided<br>*Only common tests should be described solely by name; describe more complex techniques in the Methods section.*
☐	☒	A description of all covariates tested
☐	☒	A description of any assumptions or corrections, such as tests of normality and adjustment for multiple comparisons
☐	☒	A full description of the statistical parameters including central tendency (e.g. means) or other basic estimates (e.g. regression coefficient) AND variation (e.g. standard deviation) or associated estimates of uncertainty (e.g. confidence intervals)
☒	☐	For null hypothesis testing, the test statistic (e.g. *F*, *t*, *r*) with confidence intervals, effect sizes, degrees of freedom and *P* value noted<br>*Give P values as exact values whenever suitable.*
☒	☐	For Bayesian analysis, information on the choice of priors and Markov chain Monte Carlo settings
☒	☐	For hierarchical and complex designs, identification of the appropriate level for tests and full reporting of outcomes
☒	☐	Estimates of effect sizes (e.g. Cohen's *d*, Pearson's *r*), indicating how they were calculated

Our web collection on statistics for biologists contains articles on many of the points above.

Software and code

Policy information about availability of computer code

Data collection	Untargeted metabolomics was performed using Ultrahigh Performance Liquid Chromatography-Tandom Mass Spectrometry (UPLC-MS/MS) at Metabolon, Inc. (Research Park Triangle, NC, USA). Acetylomics was performed using MS 6550 Q-TOF equipped with an Agilent nano source operated in MS-only mode.
Data analysis	Profinder V.B.10.00 software (Agilent), MetaboAnalyst version 4.0, Image J (NIH), Prism 7.0 (GraphPad)

For manuscripts utilizing custom algorithms or software that are central to the research but not yet described in published literature, software must be made available to editors/reviewers. We strongly encourage code deposition in a community repository (e.g. GitHub). See the Nature Research guidelines for submitting code & software for further information.

Data

Policy information about availability of data

All manuscripts must include a data availability statement. This statement should provide the following information, where applicable:

- Accession codes, unique identifiers, or web links for publicly available datasets
- A list of figures that have associated raw data
- A description of any restrictions on data availability

The data sets generated during and/or analyzed during the current study are available from the corresponding author, Rafael de Cabo (decabora@mail.nih.gov), on reasonable request. The metabolomics data have been deposited in the MetaboLights database (https://www.ebi.ac.uk/metabolights/) with the following accession number MTBLS2085. RAW MS data from our skeletal muscle proteome analysis have been deposited to the ProteomeXchange Consortium (http://proteomecentral.proteomexchange.org) via the PRIDE partner repository with the data set identifier PXD015070.

Field-specific reporting

Please select the one below that is the best fit for your research. If you are not sure, read the appropriate sections before making your selection.

- [x] Life sciences
- [ ] Behavioural & social sciences
- [ ] Ecological, evolutionary & environmental sciences

For a reference copy of the document with all sections, see nature.com/documents/nr-reporting-summary-flat.pdf

Life sciences study design

All studies must disclose on these points even when the disclosure is negative.

Sample size	No statistical methods were used to predetermine sample size
Data exclusions	No data were excluded from the analysis
Replication	All experiments were reproduced to reliably support conclusions stated in the manuscript. The effects of NQO1 trnsgenesis on glucose and lipid homeostasis and diet-induced liver steatosis were replicated using several cohorts at different times.
Randomization	Mice were randomly divided into experimental groups and assigned to receive either a standard or high-fat diet. No significant differences in body weight were observed at baseline.
Blinding	The investigators were blinded to group allocation during scoring of liver damage and glycogen content

Reporting for specific materials, systems and methods

We require information from authors about some types of materials, experimental systems and methods used in many studies. Here, indicate whether each material, system or method listed is relevant to your study. If you are not sure if a list item applies to your research, read the appropriate section before selecting a response.

Materials & experimental systems

n/a	Involved in the study	
[ ]	[x]	Antibodies
[x]	[ ]	Eukaryotic cell lines
[x]	[ ]	Palaeontology
[ ]	[x]	Animals and other organisms
[x]	[ ]	Human research participants
[x]	[ ]	Clinical data

Methods

n/a	Involved in the study	
[x]	[ ]	ChIP-seq
[x]	[ ]	Flow cytometry
[x]	[ ]	MRI-based neuroimaging

Antibodies

Antibodies used	The source of the antibodies used for immunoblotting were as followed: NQO1 (Abcam, cat #ab2346), mTOR (Cell Signaling, cat#2972), pSer mTOR (Cell Signaling, cat#2971), S6K1 (Cell Signaling, cat#9202), pThr S6K1 (Cell Signaling, cat#9234), RpS6 (Cell Signaling, cat#2217), pSer RpS6 (Cell Signaling, cat#2215), 4E-BP1 (Cell Signaling, cat#9644), pThr 4E-BP1 (Cell Signaling, cat#2855), IR b-subunit (Cell Signaling, cat#3025), IRS-1 (Cell Signaling, cat#3407), pSer IRS-1 (Cell Signaling, cat#2385), pSer AS-160 (Cell Signaling, cat#8730), eIF4G (Cell Signaling, cat#8701), GCLC (Abcam, cat#41463), HO-1 (Abcam, cat#13243), SOD2 (Abcam, cat#13533), acetyl-lysine (Cell Signaling, cat#13416), ACC (Cell Signaling, cat#3676), FASN (Cell Signaling, cat#3180), ACLY (Cell Signaling, cat#4332), Lipin 1 (Cell Signaling, cat#14906). The lot numbers for each of the antibodies used are unavailable.
Validation	We relied on the validation statements on the manufacturer's website for each antibody used for Western blot experiments.

Animals and other organisms

Policy information about studies involving animals; ARRIVE guidelines recommended for reporting animal research

Laboratory animals	Mus musculus, NQO1-Tg, CMVCre-Keap1flox/flox, Keap1flox/flox, C57BL6/J, males
Wild animals	The study did not involve wild animals.
Field-collected samples	The study did not involve field collected samples

Ethics oversight	All animal protocols were approved by the appropriate institutional animal care and use committee of the National Institute on Aging and the Johns Hopkins University Animal Care and Use Committee.

Note that full information on the approval of the study protocol must also be provided in the manuscript.
